# On catching the preparatory phase of damaging earthquakes: an example from central Italy

**DOI:** 10.1038/s41598-023-41625-0

**Published:** 2023-09-01

**Authors:** Matteo Picozzi, Antonio G. Iaccarino, Daniele Spallarossa, Dino Bindi

**Affiliations:** 1 University of Naples Federico II, Naples, Italy; 2 DISTAV, University of Genoa, Genoa, Italy; 3 Helmholtz Centre Potsdam, GFZ German Research Centre for Geosciences, Postdam, Germany

**Keywords:** Natural hazards, Seismology

## Abstract

How, when and where large earthquakes are generated remain fundamental unsolved scientific questions. Intercepting when a fault system starts deviating from its steady behavior by monitoring the spatio-temporal evolution and dynamic source properties of micro-to-small earthquakes can have high potential as tool for identifying the preparatory phase of large earthquakes. We analyze the seismic activity that preceded the Mw 6.3 earthquake that hit L’Aquila on 6 April 2009 in central Italy, and we show that the seismic catalog information can be transformed into features allowing us to track in a statistical framework the spatio-temporal evolution of seismicity. Features associated to foreshocks show different patterns from the background seismicity that occurred in the previous years. We show that features ensemble allows to clearly capture the activation phase of the main event. Nonetheless, foreshocks share similar clustering properties of previous seismic sequences not culminating in large earthquakes, and thus generating questions on their use as potential precursor for earthquake sequences prone to evolve into catastrophic sequences.

## Introduction

Among natural phenomena, earthquakes are one of the most impressive. Large earthquakes have strong impact on society, not only due to the loss of human lives, but also because the domino effects on our globalized society causes billions of euros of damage. Retrospective and theoretical studies on megathrust earthquakes have shown that patterns in seismicity and crustal deformation precede large earthquakes^1–8^.

The complex multi-scale generation process of large earthquakes might have different dominant features depending on the tectonic environment, such as foreshocks or slow slip events and creep phenomena^9,10^. Laboratory^11,12^ and simulation^13^ results suggest the occurrence of foreshocks being primarily related to structural and stress heterogeneities over rupture surfaces (i.e., where stress heterogeneity is higher, the foreshocks activity is the more prominent). Upscaling laboratory results to crustal faults, we can figure out that on faults with few stress heterogeneities, the preparatory process is driven by seismic and aseismic slip around a nucleation zone (i.e., aseismic model). On the contrary, on faults with diffuse stress and surface heterogeneities, we can find foreshocks triggering each other in a cascading process (i.e., cascade model). When foreshocks occur at a large and stressed asperity, the process can culminate in a large earthquake.

More in general, the large earthquake generation process is proposed being related to a progressive localization of shear deformation around a rupture zone that progressively evolves into a final rapid loading (i.e., generating the small magnitude earthquakes called foreshocks) of a crustal volume localized nearby the hypocenter of the major dynamic rupture^14^. Similar patterns of damage evolution have also been observed by studying acoustic emissions during triaxial tests on rock samples^11^, suggesting that the process generating earthquakes may be universal.

However, the non-systematic foreshocks appearance and the lack of systematic precursory patterns in seismicity and ground deformation in tectonic context different from megathrust areas are demonstrating us that the background physical processes generating large earthquakes are not fully understood yet^14^. Therefore, the prediction of large magnitude earthquakes remains an unresolved fundamental scientific question that needs to be investigated.

Thanks to the strategies implemented by the seismological community in establishing dense seismic networks to monitor regions known to be prone to large earthquakes, standardizing formats for data transmission and archiving, and creating open data repositories for sharing real-time and archived data streams, nowadays it is greatly improved our possibility to study the multi-scale (spatial and temporal) generation process of large earthquakes, and new scientific avenues have been opened. A meta-analysis of foreshocks data^15^ has shown that a preparatory phase is potentially identified when seismic catalogs are complete for at least three magnitude units less than the mainshock unit. It must keep in mind, however, that ‘foreshock’ is merely a label assigned to earthquake retrospectively.

For unveiling if preparatory processes are ongoing, instead of trying to establish whether an event is a foreshock or not, Picozzi and Iaccarino^16^ proposed to study the spatial and temporal collective behavior of small magnitude earthquakes. Applying this concept to microseismicity from The Geysers geothermal field in California, where the seismicity was characterized by features carrying information about crustal stress conditions, it was shown as a recurrent neural network could identify the preparatory phase of moderate earthquakes^16^, which raises hope for identifying the preparatory phase of future large earthquakes.

Although some success on intercepting the preparatory phase of earthquakes starts to be achieved, recent analyses on two large Italian earthquakes (i.e., the Mw 6.3 L’Aquila 2009 and the Mw 6.1 Amatrice 2016), which occurred at few tens of kilometers of distance and in the same extensional environment in the Apennines in central Italy, have highlighted important differences in their initiation^17,18^, with a clear preparatory phase identified for the former and only a long-lasting quiescence without a clear activation phase for the second. The latter examples highlight the enigmatic nature of the preparatory phase of large earthquakes, which also in case of nearby earthquakes can result be dominated by different driving mechanisms.

Although not very large in magnitude, the Mw 6.3 L’Aquila 2009 (hereinafter, AQU), Italy, earthquake is very well known within the scientific community and one of the most studied recent earthquakes in the literature (International Seismological Centre 2022, On-line Event Bibliography, 10.31905/EJ3B5LV6^19^). We find AQU interesting because previous studies proposed its preparatory phase being characterized by both the presence of foreshocks clustered near the nucleation area of the mainshock^20–23^ and by a slow-slip event^24^, despite the latter is still debated^25^.

Studies of this preparatory phase from different perspectives have highlighted a foreshocks migration towards the mainshock nucleation point^26^, *b-value* changes associated to change in the stress level on the fault^27^, changes in the elastic properties of the medium^28^, and different source properties between foreshocks and aftershocks^29^. A more detailed analysis on the spatio-temporal evolution of foreshocks source properties approaching the mainshock was made possible thank to an innovative service for the Rapid Assessment of Seismic Moment and Radiated Energy in Central Italy (RAMONES^30^), which innovatively allows studying the small magnitude seismicity by direct estimates of their seismic moment and seismic radiated energy. Picozzi et al.^17^ analyzed the temporal evolution of radiated energy and size of small magnitude earthquakes preceding AQU and showed that during the final activation phase preceding the mainshock the foreshocks had dynamic characteristics distinct from those of normal rate (background) seismicity.

The key idea here is to retrospectively study the earthquakes that have preceded AQU to explore our capability to outline the evolution of fault loading processes. The main scientific question that we face is if we can identify trends in the spatio-temporal evolution and dynamic properties of seismicity that highlight changes with respect to the crustal background activity. In other words, can we catch the preparatory phase of large magnitude events by looking at tiny earthquakes?

We reprocessed thousands of earthquake recordings that have occurred in the Apennine region since 2005, creating an innovative high-resolution seismicity catalog (see supplemental material) with information on both the spatial and dynamic properties of seismic sources. These pieces of information are transformed into features representing the spatio-temporal clustering and the dynamic characteristics of the earthquakes. Then, we analyze and exploit the estimated features to comprehensively characterize the evolving pattern of seismicity. To this purpose, we set up a probabilistic framework by which we explore the differences between the spatio-temporal and dynamic source properties of microseismicity approaching the mainshock with respect to those of the background and clustered seismicity that occurred in the past.

## Results

### Overview of seismicity features

**Figure 1:**
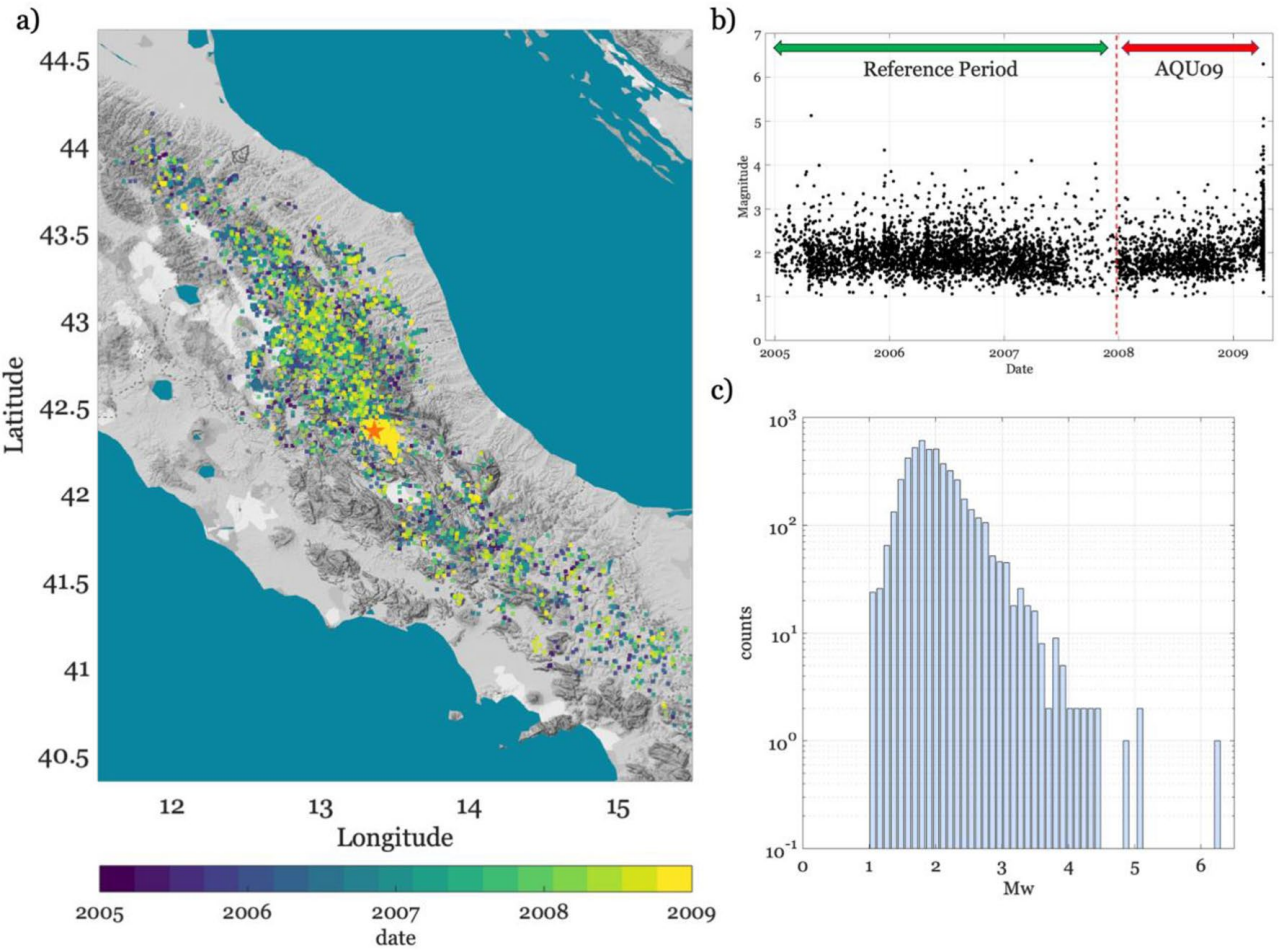
Overview of the 2005–2009 seismicity in central Italy. (**a**) location of the earthquakes colored per date of occurrence, and epicenter of the Mw 6.3 L’Aquila 2009 shown as red star. (**b**) distribution of magnitude in time. The reference and the AQU09 periods are highlighted and separated by a vertical dashed red line. (**c**) Histogram showing the distribution of Mw.

We retrieved data from publicly available databases and identified 4820 earthquakes occurred between the 1 of January 2005 until the occurrence of AQU on 6 April 2009 (Supplementary material). The earthquakes range between magnitude Mw 1 and Mw 6.3, hypocentral depths between 0.3 and 29 km, and are distributed along the central Apennines (Fig. 1). Uncertainties in event location are mostly within 1 km both horizontally and vertically (Fig. S1). We first focus on a subset of data (i.e., between 2005 and 2007) to define reference models for the spatio-temporal evolution of seismicity. Hereinafter, we refer to this subset as ‘reference period’. Considering that seismicity can be seen as composed by two main populations, that is the background seismicity associated to tectonic stress field and the clustered one having different origin (e.g., foreshocks, aftershocks, swarms), we apply a clustering analysis^31,32^ and we model the distribution with a sum of a log-Gaussian function^33^ for discriminating the two populations (hereinafter, defined as background, B, and clustered, C, Figs. S2 and S3).

**Figure 2:**
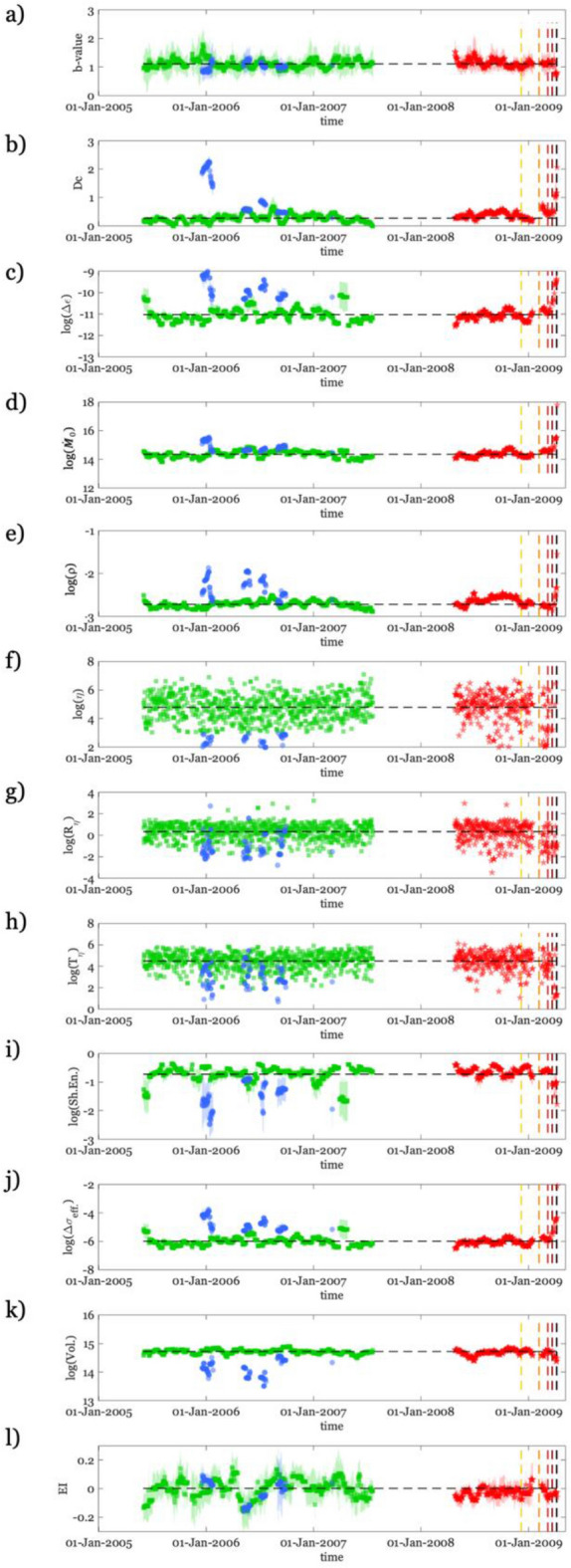
Overview of the seismic features in time. From (**a**) to (**l**) we show the evolution in time of each feature, where for *b-value*, *Dc*, $\dot{M}$_*0*_, *H*, *Δσ*_*e*_, *Δε*, and EI we show the mean value ± the standard error (vertical bar).We highlight with different colors the Background events (green), the Clustered ones (blue), and the AQU09 seismicity considered for identifying the preparatory process (red). The colored vertical dashed lines indicate 120 days yellow, 60 days orange, 30 days light red, 15 days dark red before the mainshock.

We characterize the seismicity in terms of a set of twelve physically based features varying in time and describing different aspects of the temporal and spatial evolution of seismicity: the *b-value* of the Gutenberg–Richter law^34^; the fractal dimension of hypocenters, *Dc*^35^; the generalized distance between pairs of earthquakes, *η*, and its space and time components (*Rη*, *Tη*, respectively), rate, *ρ*, and moment rate, $\dot{M}$_*0*_^36^, the Shannon’s information entropy, *H*^37^; the effective stress, *Δσ*_*e*_^38^, the volume, *V*, by the 3D convex Hull of the hypocenters in a given time window, the Kostrov strain, *Δε*^39^, and the Energy Index, EI^17^ (see “Materials and methods” section for details about the computation of seismic features). We estimate the uncertainty associated with the features by applying a bootstrap approach^40^, repeating at each time instant the features computation with 200 random sampling realizations of the original dataset with replacement. This analysis is carried out for *b-value*, *Dc*, $\dot{M}$_*0*_, *H*, *Δσ*_*e*_, *Δε*, and EI (Fig. 2), while we exclude from the bootstrap analysis the remaining five features that are function of the earthquake location and origin time only (*η*, *Rη*, *Tη*, *ρ*, and *V*). The features are computed also for the seismicity occurred from the 1 January 2008 until AQU (the latter period is indicated as AQU09). For AQU09, we do not discriminate between B and C seismicity. Indeed, we consider AQU09 as a ‘testing dataset’ and, after having characterized the B and C populations, we look for deviations in AQU09 with respect to B and C that could hints for the preparation process of the mainshock.

**Figure 3:**
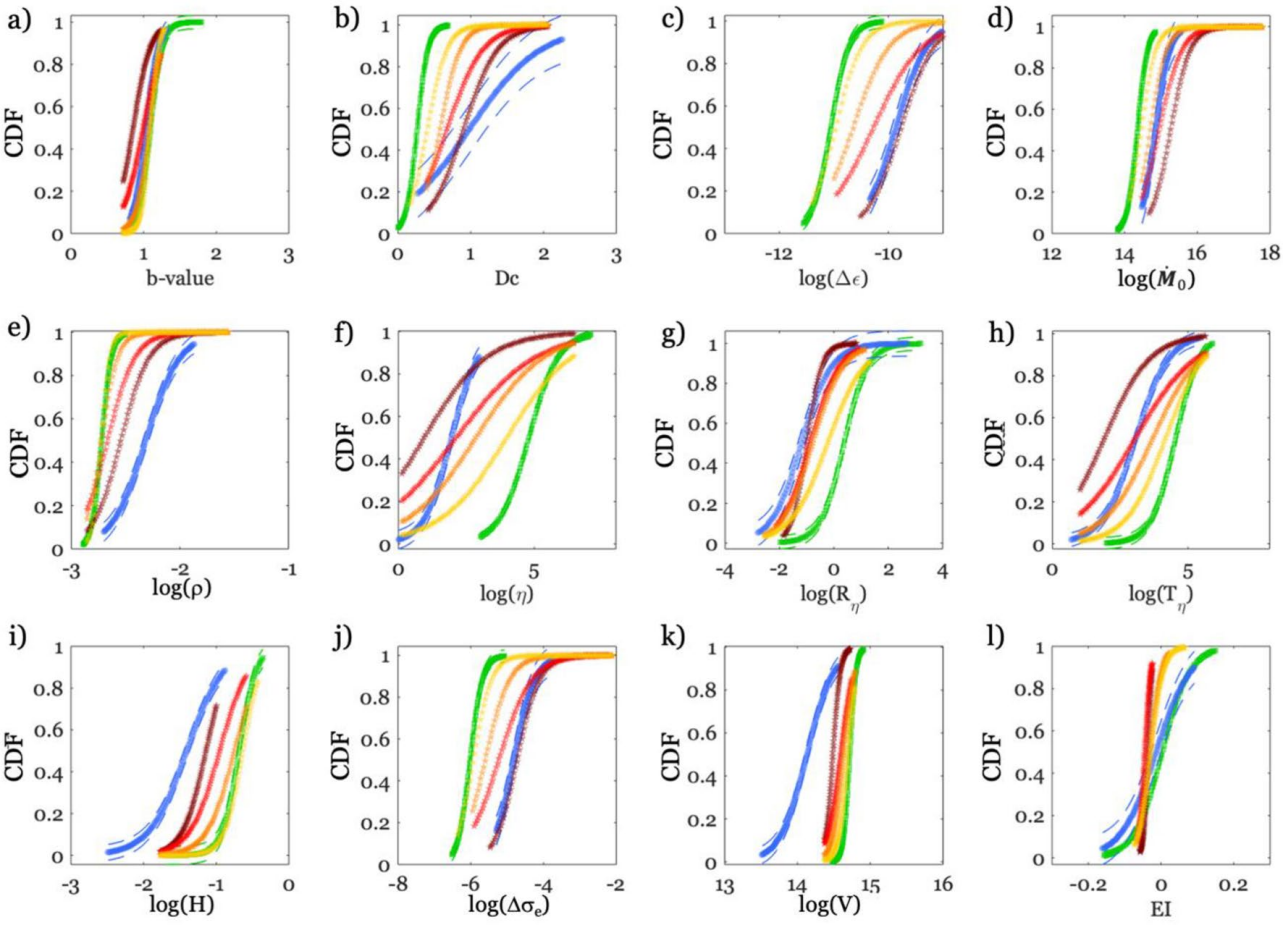
Overview of the Cumulative Density Function (CDF) computed for the different features and seismicity belonging to different periods. From (**a**) to (**l**) we show for each feature the CDF with solid lines and  ± 1 std. with dashed lines for the background seismicity in green, the clustered one in blue, and those of different time periods before the Mw 6.3 L’Aquila earthquake (120 days yellow, 60 days orange, 30 days light red, 15 days dark red).

The temporal evolution of the features shown in Fig. 2 is obtained by considering events with magnitude equal or larger than Mw 1.5. We also investigated the effect of different cutoff magnitudes (M_**cut**_) in computing the features considering the AQU09 period (i.e., Mw 1.5 Fig. S4, Mw 1.6 Fig. S5, Mw 1.7 Fig. S6, Mw 1.8 Fig. S7, Mw 1.9 Fig. S8). The gradual decrease in the number of events does not seem to influence the main trends in the temporal evolution of the features, in particular the rapid changes in the patterns during the activation phase. We compute for all the features and for different cutoff magnitudes the empirical cumulative density function, ECDF (Fig. S9) and we fit them with logistic functions (CDF in Fig. S10). The ECDF and CDF seem not affected in their trend and statistical properties with the change in M_cut_. Nevertheless, comparing the features for M_cut_ 1.5 (Fig. S4) and M_cut_ 1.9 (Fig. S8), we see that lowering the number of data (i.e., higher M_cut_) would make any conclusion about the temporal evolution of the preparatory process less robust. We also verify the implications of different area cut-offs. We thus split the background seismicity in three subsets according to the geographical distribution of the earthquakes along the Apennines (Fig. S11a, with epicenters as black points in the Northern sector, as red points in the Central one, and as blue points in the Southern one). The ECDF and PDF for the three subsets are shown in Figs. S12, S13, S14. Their overlapping indicates that for the considered areas, the features do not present statistically significant differences. The same kind of analysis for the clustered seismicity is not possible due to the lower numerosity of data in the considered period (Fig. S11b), but it will be carried out in the next future for the period 2009–2023. Concerning the reference period, looking at Fig. 2, we find that a high fraction of seismicity is classified as background (green), with respect to which many features for both the clustered one (blue) and the seismicity preceding AQU09 (red) show distinct values and trends (Fig. 2). To highlight the existence of differences between the three populations, we compute their ECDF (Fig. S15) and CDF (Fig. 3) fitting them with logistic functions (Figs. S16 and S17). For the AQU09 period, we compute the CDF considering different time periods preceding the mainshock (i.e., 120, 60, 30, 15 days before AQU that are shown as yellow, orange, red and dark red in Fig. 3, respectively).

The B and C populations show distinct CDFs trend for most of the features (Fig. 3), except for the b-value and EI for which the CDFs appear overlapping. Considering that both EI and b-value have been put in relation to the stress level on the fault^18,26^, the CDF similarity for these two features suggest that background seismicity and past seismic sequences, which did not culminate in large magnitude earthquakes, occur on small scale faults with similar properties, and that both static and dynamic stresses should reach higher values over larger spatial scales to sustain the development of large ruptures. We also find interesting the way the CDFs for AQU09 change with different time periods preceding the mainshock (from yellow to dark red in Fig. 3). We note cases where the AQU09-CDFs initially resemble the B-CDFs but progressively become like the C-CDFs (i.e., for *Δε*, *η*, *Rη*, *Tη*, *Δσ*_*e*_). This result highlights a progressive clustering of the events approaching the activation phase. All that features are indeed related to the events time and space occurrence.

Furthermore, we also see other AQU09-CDFs that are initially similar to the B-CDFs, but then becoming distinct from both B and C CDFs (i.e., *Dc*, $\dot{M}$_*0*_, *r*, *H*, *V* and *EI*, which are related to the temporal evolution of dynamic source properties).

### Temporal evolution of features

We aim to measure the temporal evolution of differences among CDF for different event populations. Our goal is to verify if deviations in source and spatial characteristics for events belonging to the AQU09 series with respect to those of background and clustered earthquakes are measurable. We thus track the relation between AQU09’s CDFs with respect to those for B and C in time.

**Figure 4:**
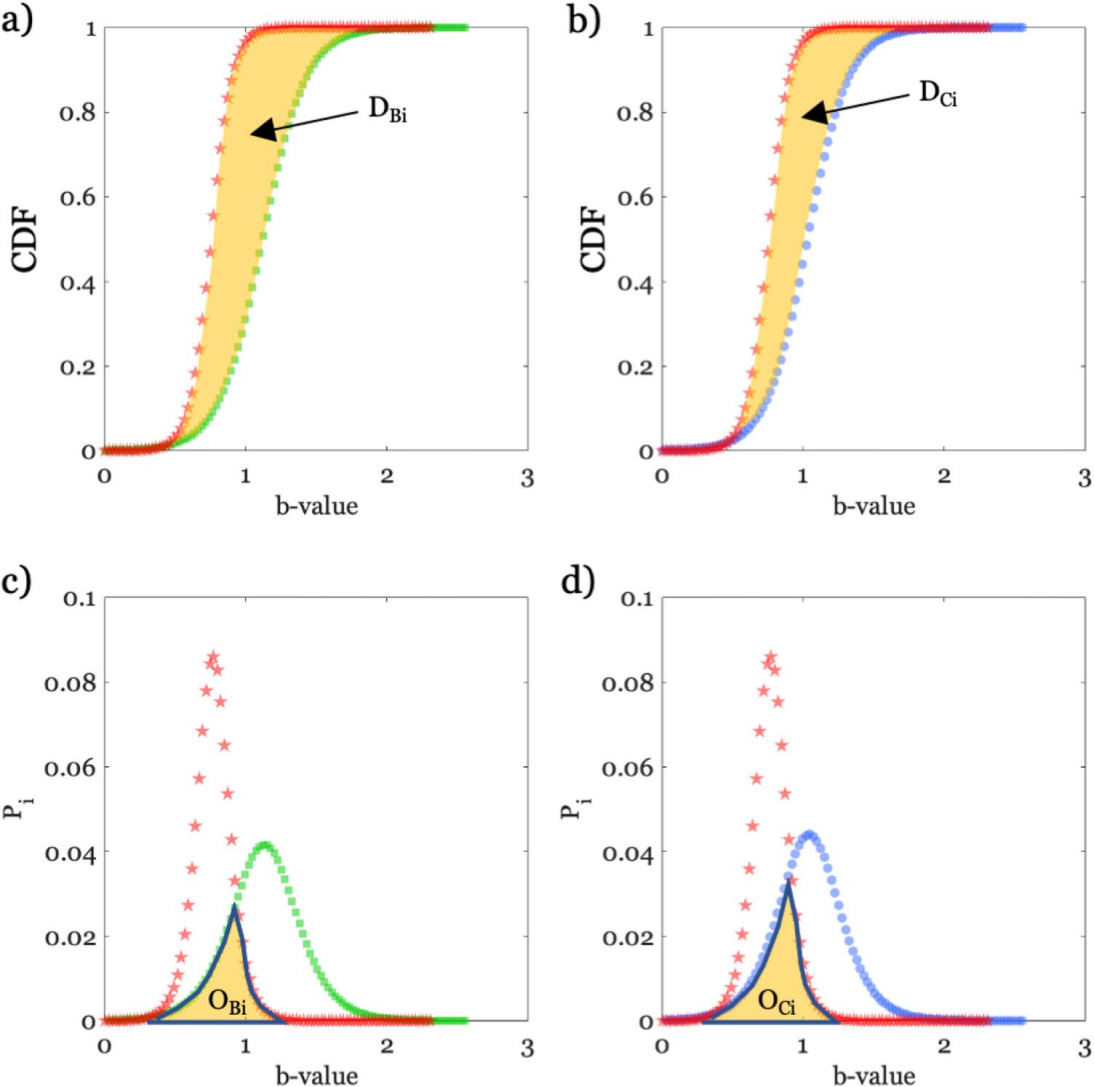
Outline of the criteria adopted to measure the distance between CDFs. (**a**) CDFs distance according to the Cramer-von Mises criterion (D_B_) with CDF relevant to the b-value for background in green and that of the AQU09 period in red at the generic time window i. (**b**) The same as (a), but with the CDF for clustered seismicity in blue (D_C_). (**c**) similar to (**a**), but considering the overlapping measure (O) between probability density functions (PDF). (**d**) The same as (**c**), but for clustered seismicity.

We explore two ways for quantifying the differences among CDFs: (1) the two sample Cramer-von Mises criterion used in statistic test^41^; (2) a distribution-free overlapping measure^42^ (as example, Fig. 4 shows the two difference measures for the b-value). In few words, by the first approach we measure the cumulative distance (D) between CDFs, while by the second one we measure the overlapping (O) between PDFs derived by CDFs (see “Materials and methods” section).

The parameters D and O are computed for all the features and considering: (i) the whole population of events for B and C; (ii) a moving window with 30 days width that moves of 1 day at time for AQU09. We thus obtain time series of D and O values with respect to both B and C (i.e., we get D_B_ and D_C_, Fig. 4a and b, and O_B_ and O_C_, Fig. 4c and d).

**Figure 5:**
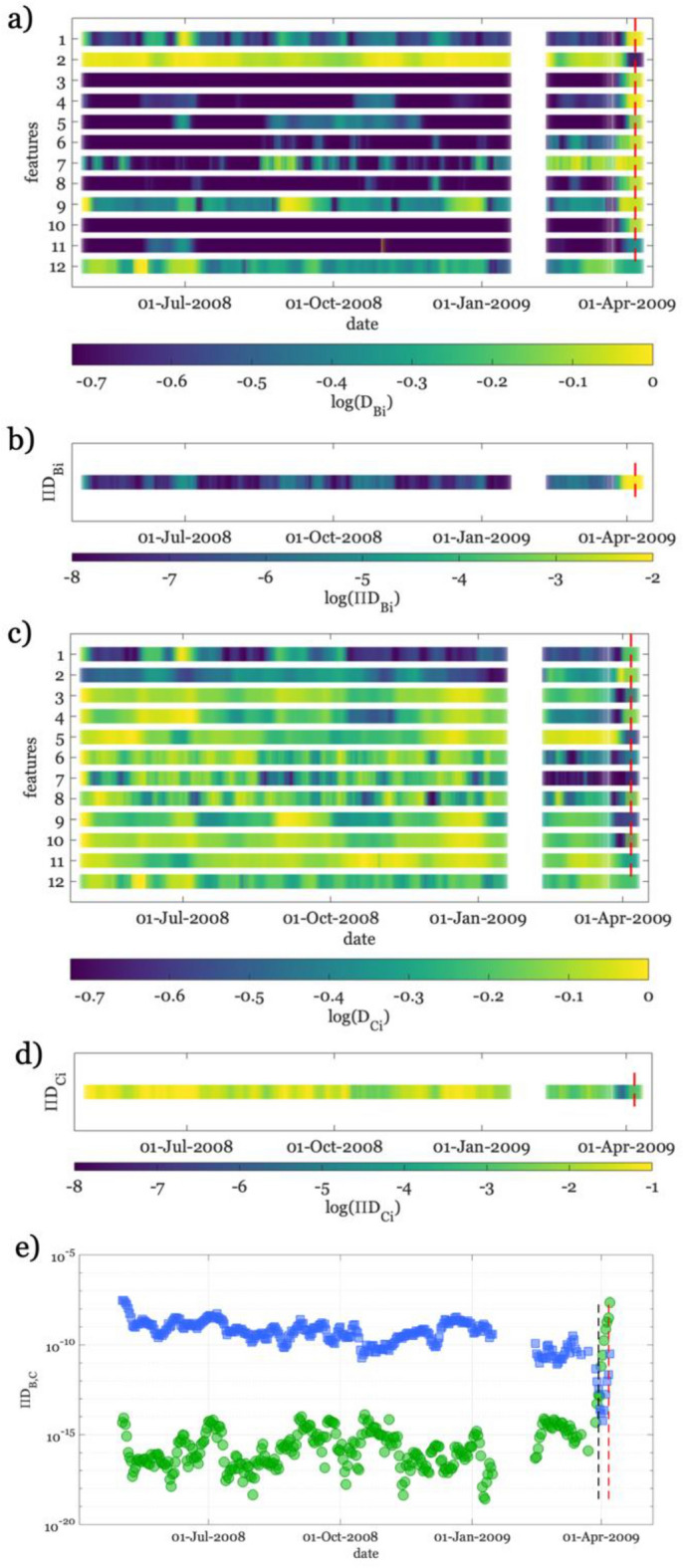
Temporal evolution of the Cramer-von Mises distance criterion for the CDFs relevant to seismic features. (**a**) For each feature it is shown the base-10 logarithm of the distance (D_B_) between the CDF for background seismicity and that for AQU09. The features are ordered as follows: 1. b, 2. Dc, 3. Δε, 4. ${\dot{M}}_{0}$, 5. ρ, 6. η, 7. Rη, 8. Tη, 9. H, 10. Δσ_e_, 11. V, 12. EI. The red dashed line represents the origin time of the mainshock. (**b** Similar to (**a**), but showing the ensemble of the CDF distances ($\prod {D}_{B}$). (**c**) and (**d**) the same as (**a**) and (**b**), respectively, but for the clustered seismicity. (**e**) Evolution of $\prod {D}_{B}$ in green and $\prod {D}_{C}$ in blue. The start of the activation phase is represented as black dashed line, while the origin time of the mainshock is shown as red dashed line.

The temporal evolution of D_B_ for all features is shown in Fig. 5a, where we plot only the last part of the reference period for better representing the preparatory phase during AQU09. During the reference period (i.e., before 2009), the features have both variable D_B_ amplitudes and incoherent trends. Conversely, during AQU09, we observe a progressive increase in D_B_ for all features while approaching the mainshock. The D_B_ trends are compliant with what we expected. At the initial stage of AQU09, the CDFs are like those of the reference period (D_B_ is very low). Conversely, D_B_ progressively increases approaching the mainshock’s activation phase because during the latter phase the CDFs become dissimilar from those of the reference period.

The ensemble of all features, $\prod {D}_{B}$, shows low values for the whole reference period and an increase before the mainshock (Fig. 5b). A closer look at the temporal evolution of $\prod {D}_{B}$ highlights that during the activation phase (i.e., from the end of March 2009), $\prod {D}_{B}$ increases of six orders of magnitude with respect to the range of values observed for the reference period (i.e., green dots in Fig. 5e).

**Figure 6:**
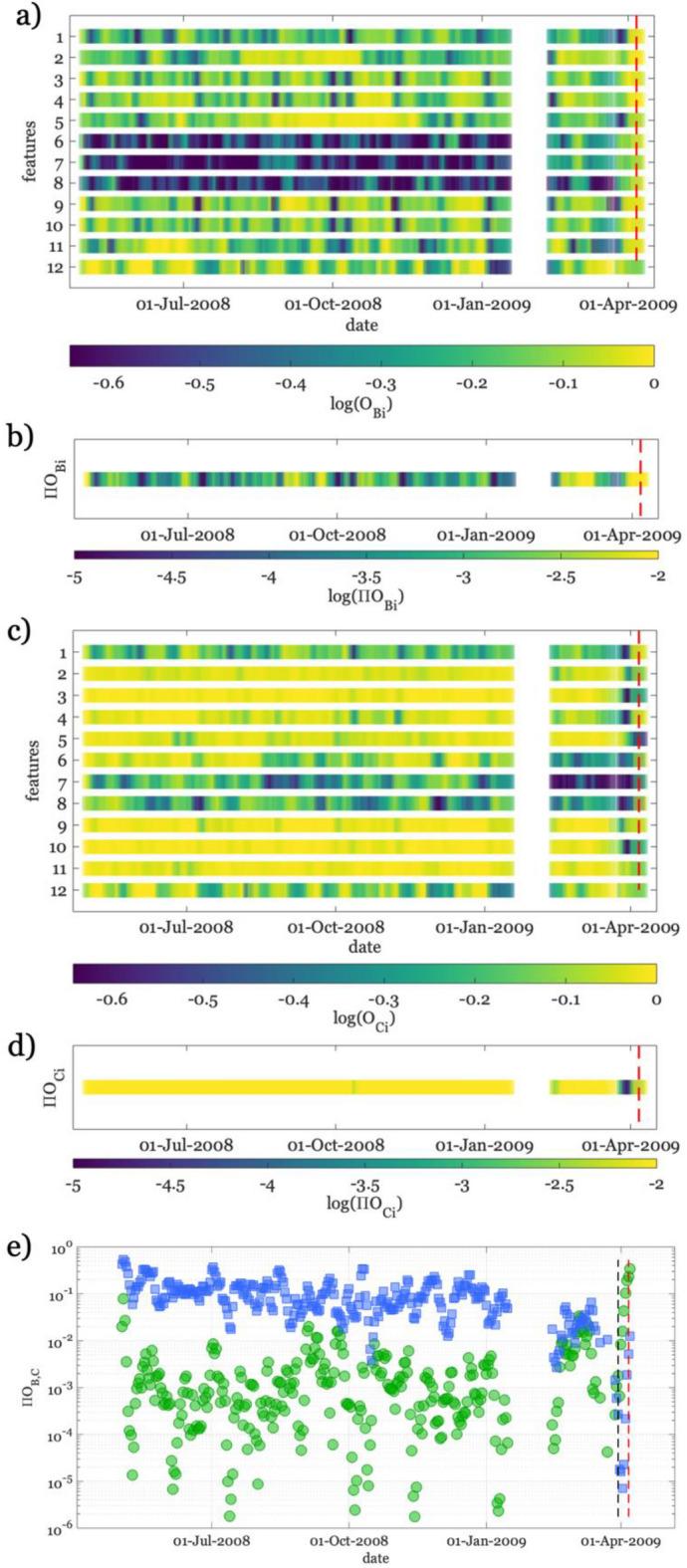
The same as Fig. 5, but for the overlapping (O) distance criterium between PDFs.

Results for D_C_ (Fig. 5c) show trends complementary to those of D_B_. D_C_ trends are in fact characterized by high values for both the reference period and the initial part of 2009. On the contrary, we observe a decrease in D_C_ for most of the features when approaching the activation phase. The high D_C_ values during the initial stage of AQU09 are clearly due to the similarity between that seismicity with the reference period one (small D_**B**_). Conversely, approaching the activation phase the CDFs for AQU09 become progressively similar to the clustered seismicity ones (small D_C_). Again, the ensemble of all features, $\prod {D}_{C}$, emphasizes the drop at the activation phase with respect to the previous period (Fig. 5d). We find intriguing the complementary trend of $\prod {D}_{B}$ and $\prod {D}_{c}$ (Fig. 5e), with the former increasing and the latter decreasing at the activation phase. We highlight that in the months preceding the activation phase neither an increase in $\prod {D}_{B}$, nor a decrease in $\prod {D}_{c}$ of a similar amount is observed. Similar results are obtained considering O_B_ and O_C_ (Fig. 6), but using them we observe a less clear distinction between the activation and the previous periods. The ensemble of all features with the overlapping parameter (i.e., $\prod {\mathrm{O}}_{B}$ and $\prod {\mathrm{O}}_{C}$) appears noisier than $\prod {D}_{B}$ and $\prod {D}_{c}$ and less efficient in discriminating the activation phase from the background seismicity (Fig. 6e). For these reasons, in the following, we will focus on $\prod {D}_{B}$ and $\prod {D}_{c}$ only.

We apply a Monte Carlo approach to assess the robustness of observed trends^17,43^. We consider the AQU09 period, and we generate for each feature 1000 random time series of values extracted from their PDF (random samples and time series are shown in Figs. S18, S19, and S20). In comparison with results of Fig. (5), we check how often: (1) the random time series have a slope during the short activation phase equal or larger than that for the reference time series; (2) if the last value is an extreme point (i.e., a maximum or a minimum). In this way, we assess the likelihood that the slope of features during the activation phase was the largest of the time series by chance, and similarly that the last value was an extreme point by chance. Since the activation phase is identified by the ensemble of features, we have also computed the mean of all computed rates (see Table S1). Table S1 shows that it is very unlikely that the observed results arise from noise, suggesting that our analysis results are robust.

### Features importance and best-set selection

**Figure 7:**
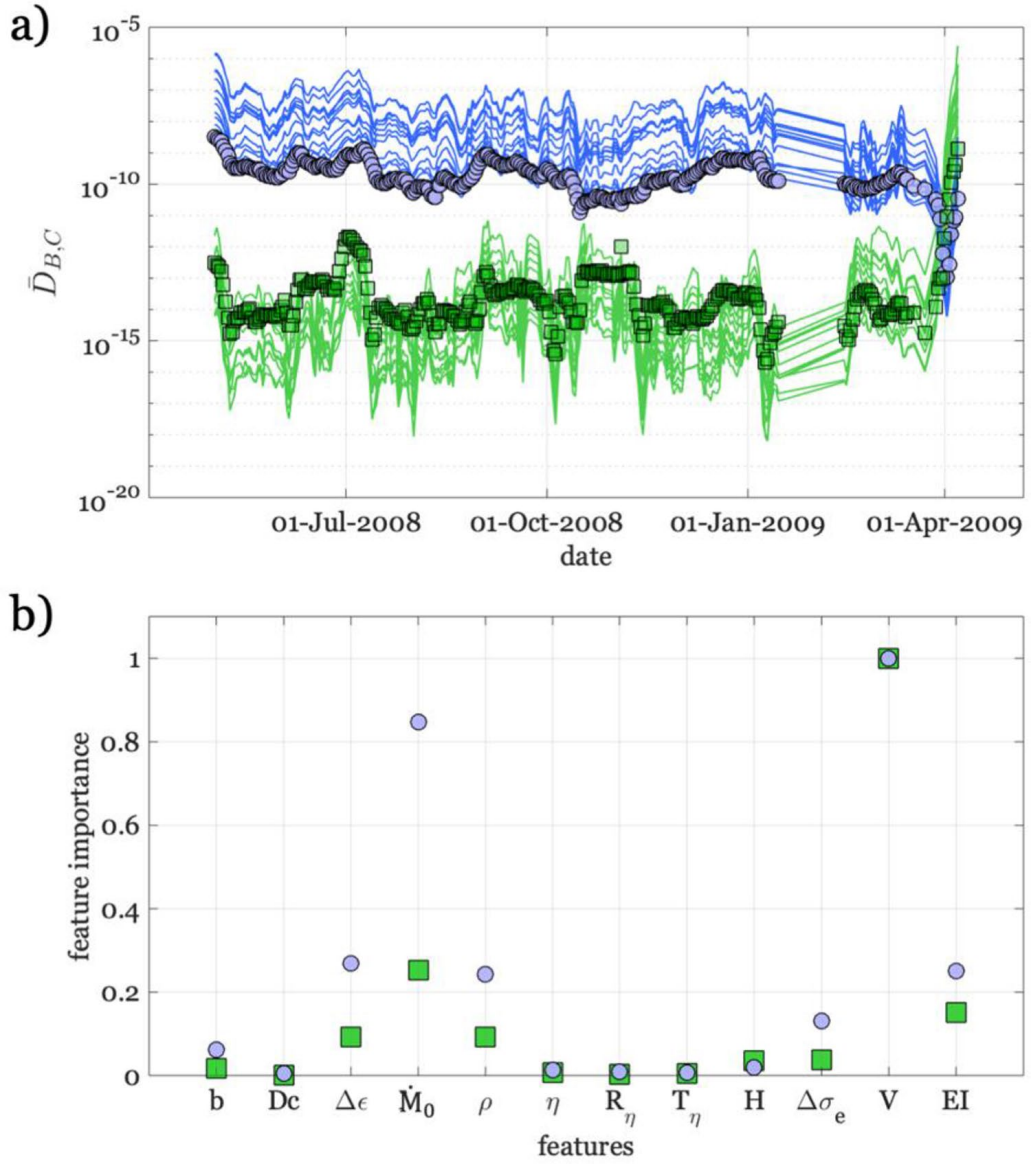
Feature importance analysis. (**a**) Comparison between D_B_ curves, whereas the reference one obtained by the ensemble of all features is shown as green squares, while those obtained neglecting one feature at time are represented as green lines. Similarly, the D_C_ for the ensemble of all features is shown as blue dots, while those eliminating one feature at time are shown as blue lines. (**b**) Normalized feature importance for D_B_, shown as green squares, and D_C_, blue dots.

We try next to understand which, among the considered features, is dominating over the others in discriminating the preparation phase. Understanding the features importance would indeed allow us to tailor workflows for the detection of the preparation phase of large earthquakes. Hence, we set up a simple feature importance analysis by measuring the root mean squared error (RMSE) between $\prod {D}_{B}$ for all the *n* features (i.e., *n* = 12) and its counterpart obtained excluding one feature at time ($\prod_{n-1}{D}_{B}$) (Fig. 7a). Intuitively, when we exclude a feature with low impact on $\prod {D}_{B}$, the two curves ($\prod {D}_{B}$ and $\prod_{n-1}{D}_{B}$) are very similar and the RMSE is low. On the contrary, excluding an important feature would determine different curves and high RMSE. Figure 7b shows the results of this analysis for both D_B_ and D_C_, where the RMSEs for the two datasets are normalized to their maximum for facilitating the comparison. Concerning D_B_, we observe that the five most important features are, in descending order V, $\dot{M}$_*0*_, EI, *ρ*, and *Δε*. When we consider D_C_, the most important features are the same, despite it changes their relative importance. Observing that the same features play a significant role for both $\prod {D}_{B}$ and $\prod {D}_{C}$ confirms us that both the characteristics with which the clustering of seismicity occurs and the change in dynamic properties of the foreshocks with respect to the background activity are key elements of the activation phase of the L’Aquila earthquake.

**Figure 8:**
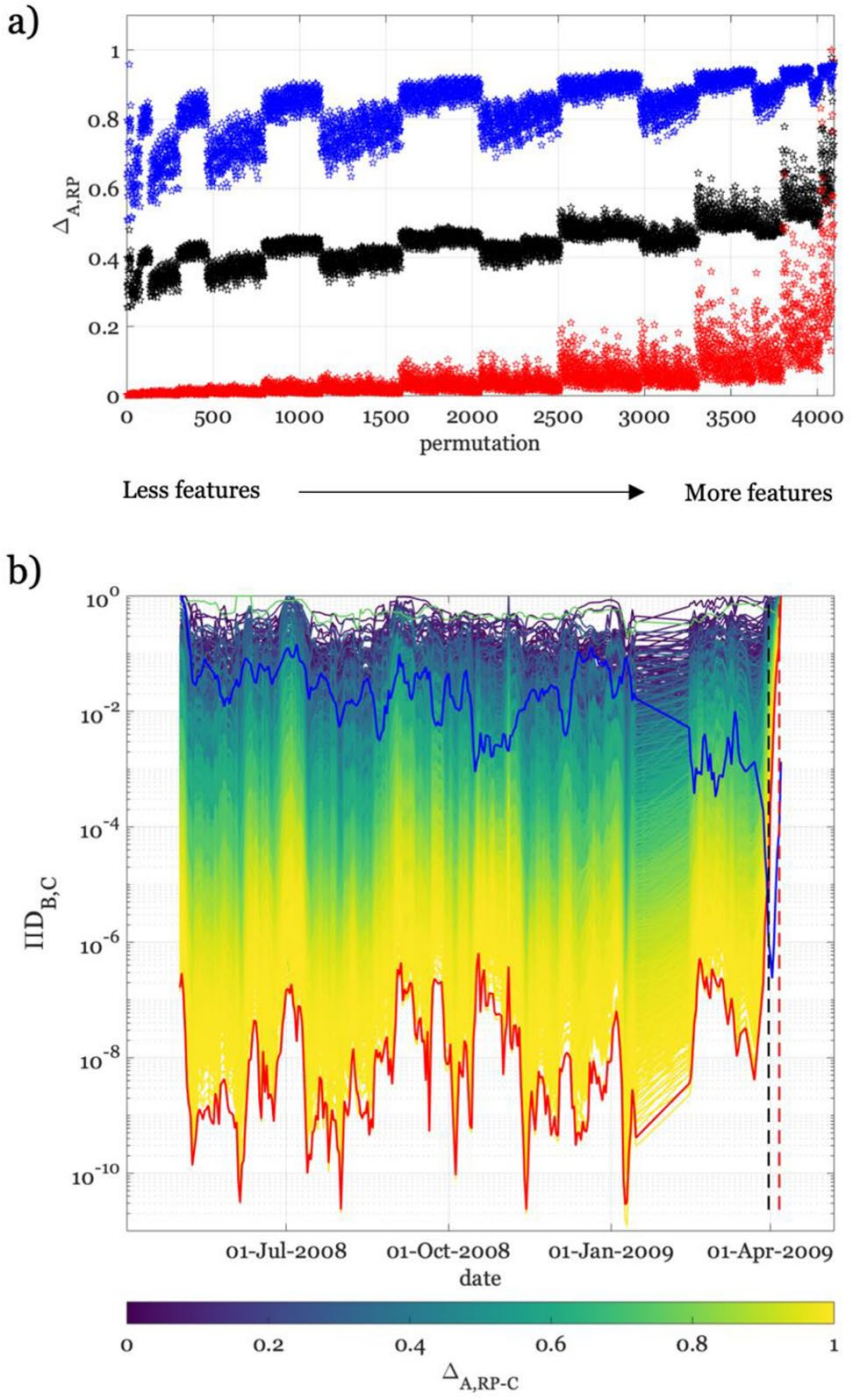
Analysis for identifying the set of features maximizing the difference between activation phase and background seismicity. (**a**) Here, we represent, as function of different number and combination of features, the difference between the ensemble of features during D_B_ in the activation phase and in the reference-period is shown as red stars. We also plot the distance for D_C_ considering the clustered seismicity and the activation phase, which is shown as blue stars. The average of the two measures is shown as black stars. (**b**) D_B_ curves colored per the difference parameter Δ_A,RP-C_. The curves corresponding to the set of features that at the same time maximize the distance between the activation phase and the background seismicity in D_B_ and minimize that one between the activation phase and the clustered seismicity in D_C_ are shown as red and blue curves, respectively.

Besides a classification of the features importance, we also search for a set of features that allows to maximize the difference between the activation phase (i.e., up to 6 days before the mainshock) and the reference period (Δ_A,RP_) in terms of D_B_, and, at the same time, to minimize the one between the same time periods in terms of D_C_. This analysis aims to verify if it is possible to separate the features in two groups with different sensitivity to the clustering phenomena and to the change in dynamic properties. One fascinating hypothesis is that features independent from the spatial organization of the seismicity but sensitive to the activation phase would be informative of the stress level on the fault, and thus about the future size of the main event. Figure 8a shows the two distances Δ_A,RP_ (i.e., red for D_B_ and blue for D_C_) with respect to the permutations (i.e., a different number and combination of features), where with the increase of permutation number does also increase the number of features considered. We combine the two Δ_A,RP_ measures assigning to them the same importance (i.e., we use their mean, Δ_A,RP-C_, shown as black dots in Fig. 8a). Figure 8b shows all the curves colored per Δ_A,RP-C_, where D_B_ (red) and D_C_ (blue) for the best combination of features are highlighted (i.e., the case where ten over twelve features are used). Only the fractal dimension (2) and the energy index (12) are excluded from this optimal set of features. These results also suggest that there is no feature able to intercept the activation phase alone. Of course, this conclusion is related to the features considered here; while, hopefully, looking at other foreshocks properties would unveil new information.

## Discussion

The short-term activation of the 2009 L’Aquila earthquake falls into a well-known precursory pattern of large earthquakes^44^. This earthquake seems to comply rather well with the model proposed by Kato and Ben-Zion^14^. Despite our features-based methodology seems potentially useful to intercept the preparatory phase of events behaving as the 2009 L’Aquila earthquake, it is worth mentioning that different precursory patters have been observed in the past^8, 45^ (e.g., quiescence, accelerating seismic release, doughnut, and event migration inward and outward the nucleation area). Differences in the preparatory phase for the 2009 L’Aquila and the nearby 2016 Amatrice earthquakes highlight that crustal processes leading to large earthquakes are influenced by unforeseeable combination of heterogeneous fault properties, stress interactions and crustal fluids. For instance, it was proposed that the high-pressure deep CO_2_-dominated fluids along the central and southern Apennines^46,47^ is responsible for the nucleation of large earthquakes in Italy (e.g., the 1997 Colfiorito, the 2009 L’Aquila, and the 2016 seismic sequences^48,49^).

We cannot generalize our results and it is certainly crucial to carry out further also considering different tectonic context (e.g., among others the Kumamoto, 2016, Valparaiso, 2017, and Ridgecrest, 2019 earthquakes).

Our statistical approach, which is still merely a preliminary workflow, can be easily extended for integrating features derived from other geophysical measurements repeated in time (e.g., geodetic deformation, geochemical parameters). Therefore, we believe that it could become a perfect tool for the multi-disciplinary study of active fault systems at near fault observatories. A similar principle was applied to forecast strong aftershocks in earthquake clusters in northeastern Italy and western Slovenia^50^. Our understanding of the processes occurring in the crust is inevitably hampered by the impossibility of collecting measurements directly within the seismogenic volumes^51^. This is certainly one of the most important limitations we must face while attempting to identify the preparatory process of large earthquakes. To overcome this obstacle, besides establishing denser seismic networks nearby active faults, we must boost up the use of microearthquakes as indicators of the mechanical state of the crust^18,52^. A key advantage of microearthquakes is that they are too small to interact with each other and therefore contribute little to crustal deformation^53^, but their properties and distribution in time and space are sensitive to stress changes. Therefore, in the framework of the Kato and Ben-Zion’s model^14^, microearthquakes can help to intercept and to better understand processes occurring within the crust. The increasing availability of augmented seismic catalogs, especially along deep transition zones of megathrusts, or acoustic emissions in case of laboratory stick–slip experiments, is pushing the scientific community in mining data across wide spatio-temporal scales, from which it emerges that the spatio-temporal evolution of microseismicity/acoustic emissions provide information on seismic friction and coupling. The latter pieces of information resulted useful to also predict the occurrence time of laboratory earthquakes, seismic tremor, and slow slip events^54–56^.

Previous retrospective studies investigated specific aspects of the preparation phase of the L’Aquila earthquake, and they highlighted: b-value changes^25^; foreshocks migration towards the nucleation area of the mainshock^24,25^; different source properties between foreshocks and the preceding background seismicity^17^. Differently from previous studies, we have set up a workflow that allows to both characterize the evolution of the microseismicity spatio-temporal characteristics and source properties and then to compare their statistical properties with those of background and clustered seismicity. Instead of focusing on the properties of each single events, we use a probabilistic framework working on subgroups of events evolving in time. Indeed, we believe that only studying the collective spatial patterns and properties of earthquakes we will be able in future to get hints of the proximity to a large rupture (i.e., as recently done in stick–slip laboratory experiments^57,58^).

Our results highlight that not only a feature like the b-value, which is well-known being representative of the stress conditions associated to ruptures^59^, but also other features (e.g., the fractal dimension, the 3D convex Hull volume of the hypocenters, the seismic rate and moment rate, just to mention a few of them) seem able to mark the deviation from the features trend observed during the interseismic period, especially when their ensemble is considered. While the activation phase start (~ 1 week before AQU) is well intercepted, it is worth mentioning that our feature-based probabilistic framework seem rather blind to the long-term variations in seismicity occurrence and spatial organization observed by Sugan et al.^26^. When we consider the 2 months before the mainshock, some of our features seem to delineate a population distinct from that of background seismicity (e.g., the Shannon’s information entropy, *H*, the effective stress, Δσ_e_, and the Kostrov strain, Δε, in Fig. 3). However, further studies are necessary to clarify if one of these features, or their ensemble, can highlight the presence of such long-term deviation from the background seismicity.

An issue limiting the power of the adopted approach is the minimum magnitude considered in the event selection (i.e., Mw = 1.5), which limits the number of events usable to characterize the spatio-temporal distribution and properties evolution of the microseismicity. The used threshold in magnitude is also due to difficulties in estimating the seismic radiated energy for small magnitude events (used for computing the energy index and the Shannon’s entropy). Hence, further efforts will focus on strategies to characterize smaller magnitude events.

We have shown that it is possible to identify deviations of seismic activity from a background level. Hence, studying features describing different physical processes and monitoring their spatio-temporal evolution might represent a powerful tool for intercepting hints of large earthquake preparatory processes. However, we must recognize that our results suggest that we are not able to foresee towards which state the system is evolving (large earthquake vs. seismic sequence with low magnitude earthquakes). In respect to this issue, our level of knowledge seems still limited. For this reason, we believe that it is necessary to carry out systematic studies of source properties for foreshocks and seismic sequences to identify if differences among these two seismic populations exist, and to investigate if some of the features bring information on the size of the future event.

## Materials and methods

### Dataset and features computation

To characterize the spatio-temporal and source properties evolution of seismicity, we use information extracted from a seismic catalog including 4820 earthquakes occurred in central Italy between the 1 January 2005 until the occurrence of AQU on 6 April 2009 (supplementary material S1). The catalogue includes for each earthquake information about the origin time, the hypocentral location, local magnitude, seismic moment and radiated energy (supplementary material S1). The latter parameters are estimated following the RAMONES procedure (http://www.distav.unige.it/rsni/ramones.php^30^), which exploits continuous data streams stored in free repositories (i.e., ORFEUS-EIDA, IRIS, DPC).

#### b-value

The b-value is estimated analyzing the frequency-magnitude distribution by the Gutenberg–Richter law^34^

1 $${\text{logN }} = {\text{ a }}{-}{\text{ b}}\cdot{\text{Mw}},$$

where N is the cumulative number of earthquakes, a and b values are parameters describing the productivity and relative event size distribution). The b-value is obtained by the maximum likelihood approach^60^). Together with the b-value, we retrieve the simultaneous estimate of the completeness magnitude Mc, which is useful to estimate some of the following features.

#### Fractal dimension

The fractal dimension of earthquake hypocenters, Dc, is computed applying the correlation integral method^35^:

2 $${D}_{c}=\underset{r\to 0}{\mathrm{lim}}\frac{\mathrm{log}{C}_{r}}{\mathrm{log}(r)},$$

where r is the radius of a sphere of investigation and Cr is the correlation integral:

3 $${C}_{r}= \underset{n\to \infty }{\mathrm{lim}}\frac{1}{{n}^{2}}\sum_{i=1}^{n}\sum_{j=1}^{n}H(r-\left|{x}_{i}-{x}_{j}\right|),$$

with n indicating the number of data in the analysis window (i.e., n = 200 events), x the hypocenter coordinates, and H the Heaviside step function H(x) = 0 for x ≤ 0 and H(x) = 1 for x > 0. Finally, the fractal dimension Dc is estimated as the slope of the best-fit straight line of Cr versus the distance r in a bi-logarithmic diagram.

#### Moment rate

We compute the moment rate $\dot{M}$_*0*_^61^ as follows:

4 $${\dot{M}}_{0}=\rho {M}_{o}A\frac{b}{1.5-b}\left[{10}^{(1.5-b)({m}_{max}-{m}_{0})}-1\right],$$

where ρ is the seismic rate of events larger than Mc, M_0_ and m_0_ are the seismic moment and magnitude corresponding to Mc, A is the area of finite extension including the events (in km^2^), m_max_ is the largest magnitude in the catalogue.

#### Seismic rate

We compute the seismic rate ρ^57^ considering the number of events ΔN with magnitude larger than the completeness magnitude, Mc, that occurred in a time window ΔT in areas of finite extension A

5 $$\rho =\frac{\Delta N}{(\Delta T\cdot A)},$$

where ρ represents the events per day per square kilometers (eqks./(day·km^2^)).

#### Effective stress drop of earthquake clusters

Following Fisher and Hainzl^38^, we compute the effective stress drop of earthquake clusters as follows:

6 $$\Delta {\sigma }_{e}= \frac{7}{16}\frac{\sum {M}_{0}}{{R}^{3}},$$

where R is the radius of the 3D convex Hull of the hypocenters and ΣM_0_ is the sum of seismic moments in a given time window.

#### Nearest-neighbor distance, η, rescaled distance, R_η_, and time, T_η_

The nearest-neighbor approach^31,32^ computes the generalized distance between pairs of earthquakes, η, from the analysis of the time–space distances between pairs of earthquakes. The parameter η is derived computing the distances in time (i.e., rescaled time, T_η_) and space (i.e., rescaled distance, R_η_) between an event i and its parent j normalized by the magnitude of the parent event as follows:

7 $${T}_{ij}={t}_{ij}{10}^{-b{m}_{i}/2},$$

8 $${R}_{ij}={\left({r}_{ij}\right)}^{{D}_{c}}{10}^{-b{m}_{i}/2},$$

where m is the magnitude (Mw), b is the parameter of the Gutenberg–Richter law, t is the earthquake intercurrence time, r is the earthquake distance, and D_c_ is the fractal dimension. The values of b and D_c_ are changed according to the estimates obtained for the considered window of events.

Finally, η is defined as:

9 $$log{\eta }_{ij}= log{R}_{ij}+ log{T}_{ij},$$

In our work, η, R_η_ and T_η_ are considered as features.

#### Shannon’s information entropy

The Shannon entropy^37^, also known as information entropy, provides a measure of the disorder level in a system. We compute the Shannon entropy using a regular 2-D grid (each 1.5 km × 1.5 km).

We compute the Shannon entropy as

10 $$H= - \sum_{k=1}^{m}\frac{{e}_{k}}{{E}_{R}}\left[\mathrm{ln}\frac{{e}_{k}}{{E}_{R}}\right],$$

where e_k_ represents a fraction of the total seismic energy E_R_ radiated within the kth cell. We rely here on seismic radiated energy from RAMONES. The e_k_/E_R_ ratio is assumed to represent an empirical approximation of the probability of the seismic energy radiated in the kth cell, P_k_(E_R_), with respect to the total radiated seismic energy, conditioned on the total energy radiated.

Equation (10) can therefore be rewritten as

11 $$H= - \sum_{k=1}^{m}{P}_{k}\left({E}_{R}\right)\left[\mathrm{ln}{P}_{k}\left({E}_{R}\right)\right],$$

Therefore, computing H at a given temporal interval consists of summing up the e_k_/E_R_ ratio for the entire grid. To allow comparison between different time intervals and to ensure unity total probability, H is generally normalized to the equipartition entropy HE, which corresponds to the case where E_R_ is uniformly distributed in the cells (i.e., given by the sum of E_R_ divided by the number of cells). The normalized information entropy h = H/HE ranges between 1 and 0, which correspond to the total disorder of the system and the extreme concentration of events, respectively.

The Shannon entropy concept provides hence a useful quantification of the system predictability; where h = 0 suggests the highest level of predictability and h = 1, on the contrary, suggests high disorder and low predictability.

#### Volume

We estimate the volume, V, as the 3D convex Hull of the hypocenters in a given time window.

#### Kostrov strain

The Kostrov strain *Δε*^39^ is computed as follows:

12 $$\Delta \varepsilon =\frac{\sum {M}_{0}}{2\mu V},$$

where μ is the rigidity module, V the volume defined by the hypocenters and ΣM_0_ is the sum of seismic moments of earthquakes in a given time window.

#### Energy Index

The Energy Index^17^, EI, is derived considering seismic moment, M_0_, and radiated energy, E_R_, estimates obtained by RAMONES. The seismicity that occurred over the period 2005–2007 and consisting of 461 earthquakes has been used to calibrate a reference scaling model between the base-10 logarithm (indicated as ‘log’) of M_0_, and E_S_^17^.

EI is then computed for new earthquakes as:

13 $${\text{EI }} = {\text{log}}\left( {{\text{E}}_{{\text{S}}} } \right) \, {-}{\text{ log}}\left( {{\text{E}}_{{{\text{St}}}} } \right)$$

where E_S_ is the experimental estimate for new earthquakes and E_St_ is the energy value derived from the median E_S_-to-M_0_ reference scaling model for the M_0_ of the experimental earthquakes. Positive EI values indicate that an earthquake has radiated with respect to the reference model more energy per unit-slip and unit-area (i.e., per seismic moment, M_0_) than expected. On the contrary, negative EI values are associated with earthquakes showing an excess of slip or larger rupture area and lower stress drop with respect to what expected from the reference model.

### Outline of the analyses

We divide the seismic catalog including 4820 earthquakes (supplementary material S1) occurred in central Italy between 1 January 2005 until the occurrence of AQU on 6 April 2009 as follows: (i) we consider earthquakes occurred between 2005 and 2007 (i.e., referred as ‘reference period’) for calibrating the spatio-temporal and source dynamic properties of events belonging to the background, B, and clustered, C, seismicity (supplementary materials S2 and S3). Therefore, we split the data in two subsets (B and C); (ii) a third subset of data includes the events from 1 January 2008 and until the Mw 6.3, 2009 L’Aquila earthquake (supplementary material S4).

The discrimination between B and C seismicity in the reference period is carried out by computing the generalized distance, η, of the events, and modeling the η distribution with the sum of two log-Gaussian functions^29^ (Fig. S2). By using a threshold η value (i.e., η = 3) for discriminating between B from C (Fig. S3), we implicitly accept that we will include a small portion of events belonging to one population into the other.

The third population of events includes all the earthquakes occurred after the 1 January 2008. For the latter, we do not discriminate between background and clustered seismicity because we are interested to analyze al the data for assessing the information provided by features about the preparatory phase of the mainshock.

We characterize the three populations (B, C and AQU09) computing the features on windows of events with fixed length (i.e., l = 30 days). For each window, the feature values are assigned to the end time of the last day in the window. Windows move of 1 day at time, hence each feature represents a time series. Windows with less than 50 earthquakes are discarded.

We then compute for each feature the Empirical Cumulative Density Function, ECDF, for both the B and C populations (Fig. S15), and we use a generalized linear regression model to fit them with a logistic function (Figs. S16 and S17). The latter analysis is also repeated for the features belonging to the AQU09 period, but in this case considering limited sets of data. For instance, for the results shown in Fig. 3, we adopted time windows of different lengths (i.e., 120, 60, 30, 15 days preceding the mainshock). Differently, during the temporal analysis of features, and their ensemble, shown in Figs. 5 and 6, we used windows with length 15 days moving 1 day at time.

### Supplementary Information

Supplementary Information.

## Data Availability

We used data and information retrieved from ORFEUS-EIDA (https://www.orfeus-eu.org/data/eida/), IRIS (https://www.iris.edu/hq/) and DPC (http://ran.protezionecivile.it/EN/index.php). We used data mainly from networks IV (10.13127/SD/X0FXnH7QfY), IT (10.7914/SN/IT) and MN (10.13127/SD/fBBBtDtd6q). Supplemental material includes 8 Figures.
