# Supplementary material for: On catching the preparatory phase of damaging earthquakes: an example from central Italy
Source: Sci Rep. 2023 Sep 1;13:14403. doi: 10.1038/s41598-023-41625-0 (PMC10474115; doi:10.1038/s41598-023-41625-0)
Supplement: Supplementary file 1 — Supplementary Information. [file 41598_2023_41625_MOESM1_ESM.pdf]

Supplementary Materials for

On catching the preparatory phase of damaging earthquakes: an example from central Italy

Matteo Picozzi*, Antonio G. Iaccarino, Daniele Spallarossa, Dino Bindi

*Corresponding author. Email: matteo.picozzi@unina.it

This PDF file includes:

Figs. S1 to S20 and Table S1

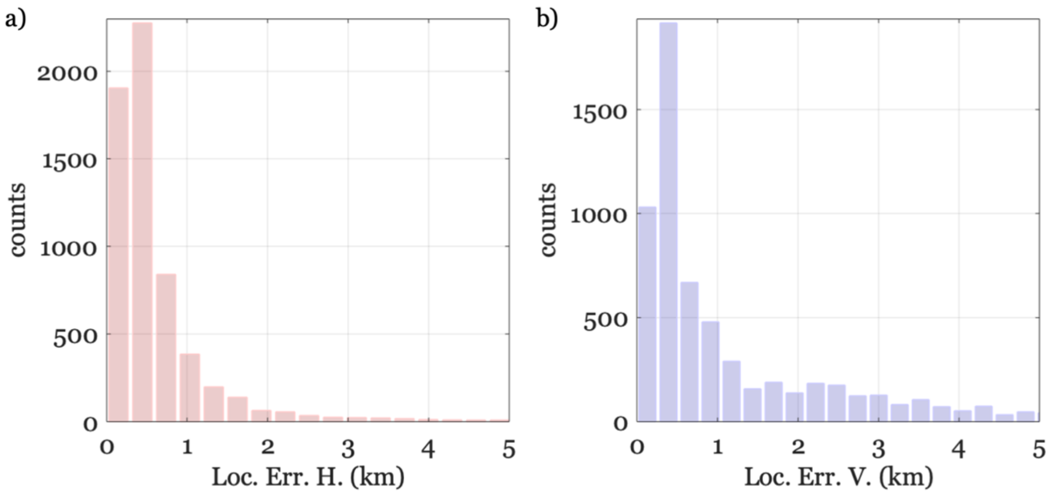

Figure S1. Histogram showing the distribution of location error for the horizontal (Left panel) and vertical (right panel) directions.

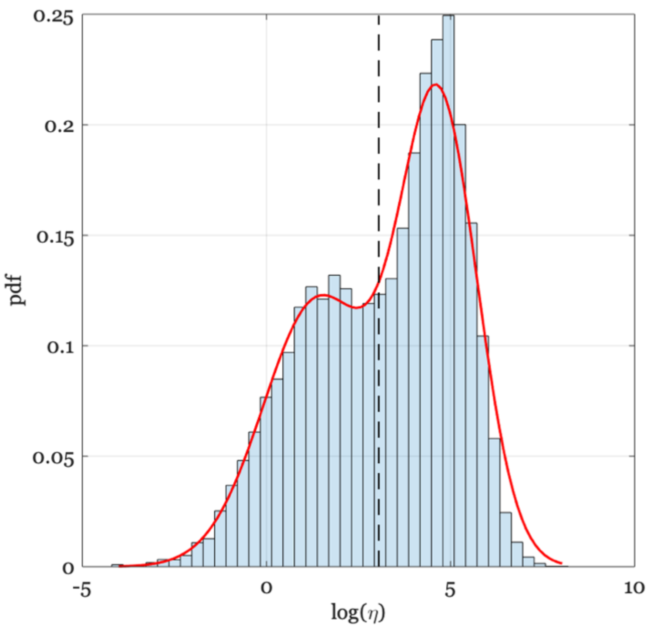

Figure S2. Histogram of the nearest-neighbor distance η, which is modeled by the sum of two log-Gaussian function (red line). Threshold h value considered for discriminating the two populations (dashed black line).

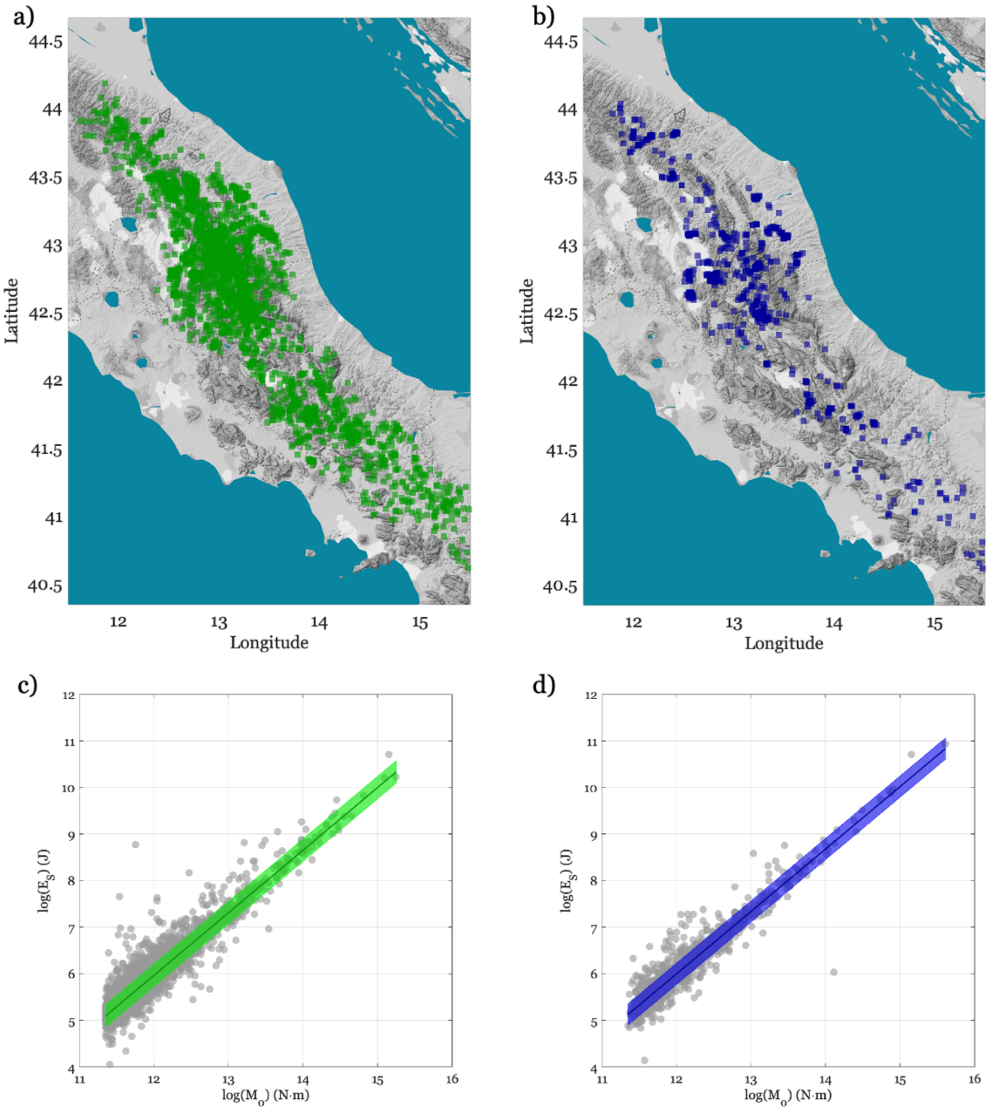

Figure S3. (**a**) Distribution of events belonging to background. (**b**) The same as (a), but for the clustered seismicity. (**c**) Radiated seismic energy scaling over seismic moment for background seismicity. (**d)** The same as (c), but for the clustered seismicity.

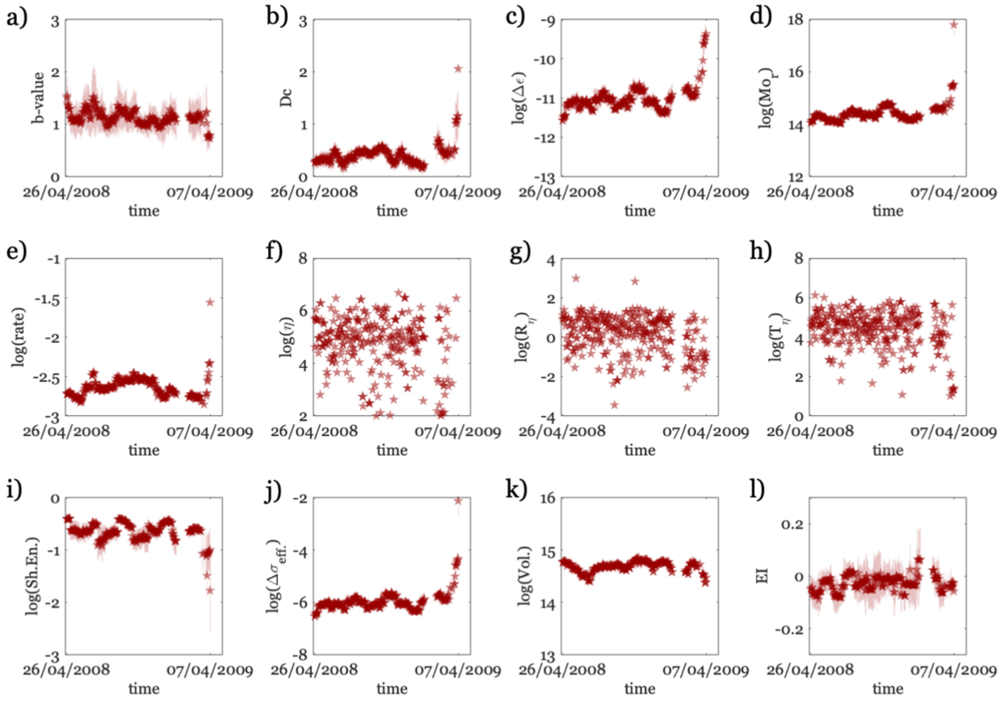

Figure S4. Features for AQU09 computed considering a cutoff magnitude equal to Mw 1.5

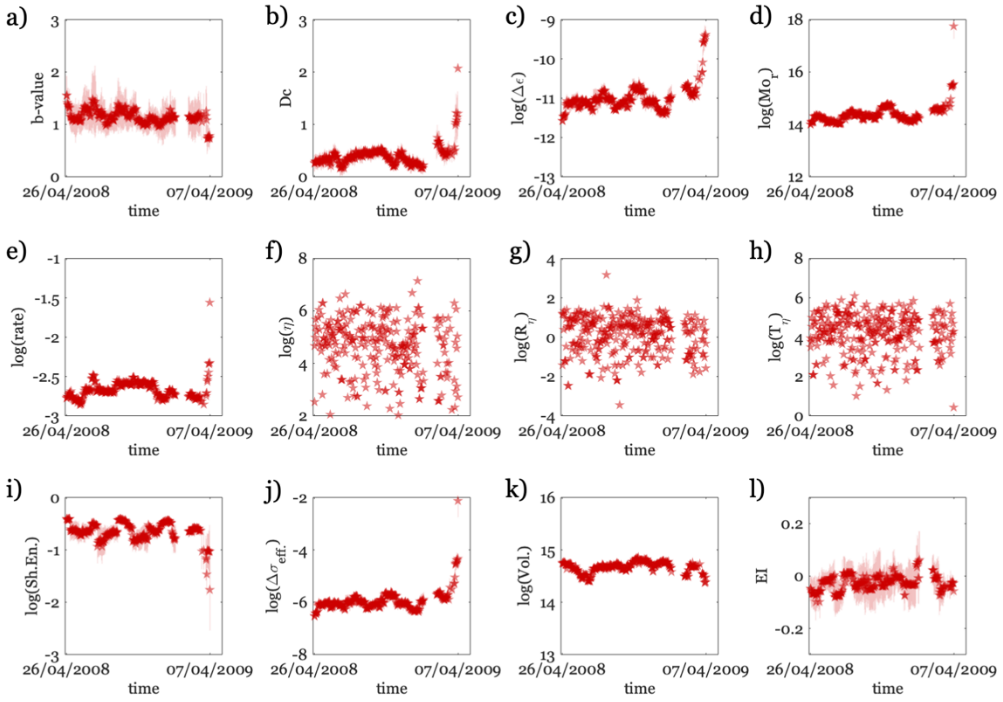

Figure S5. The same as Figure S4, but considering a cutoff magnitude equal to Mw 1.6

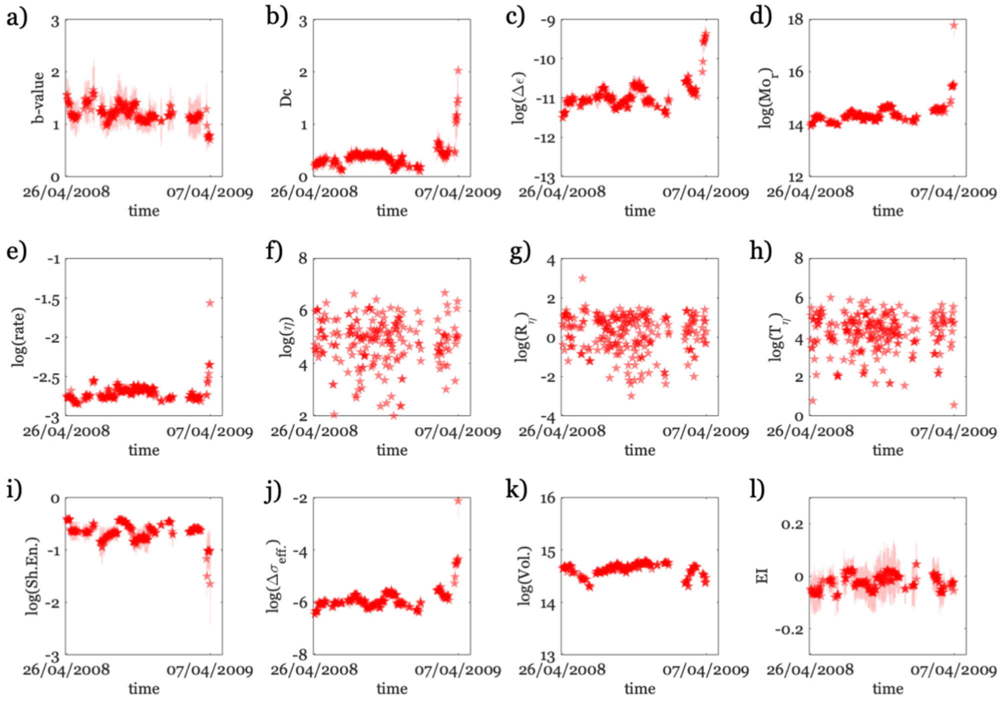

Figure S6. The same as Figure S4, but considering a cutoff magnitude equal to Mw 1.7

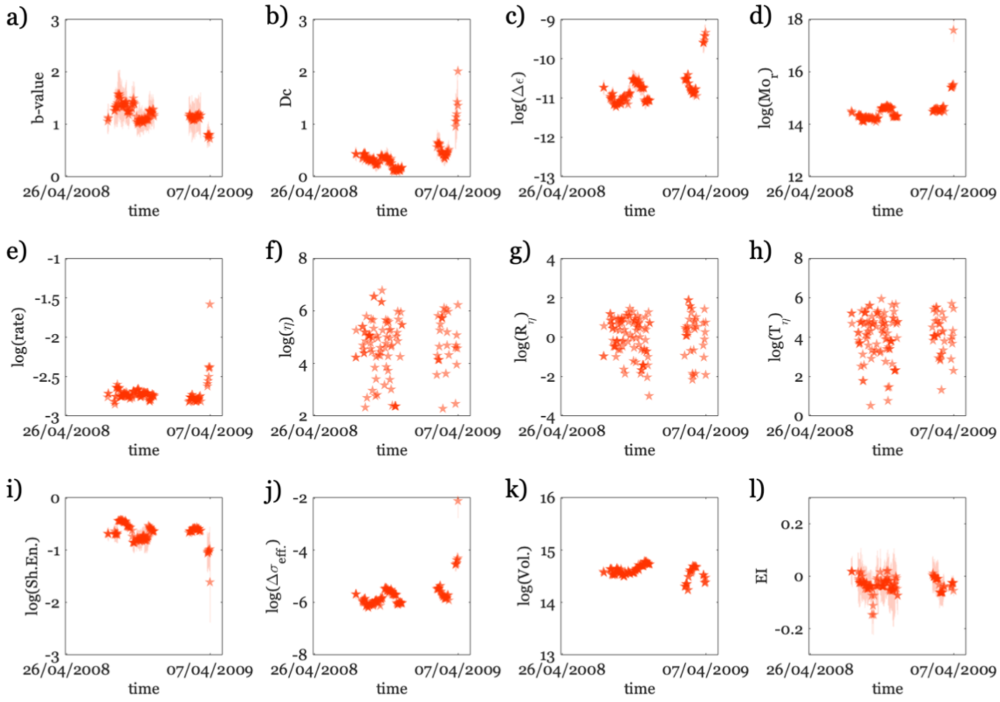

Figure S7. The same as Figure S4, but considering a cutoff magnitude equal to Mw 1.8

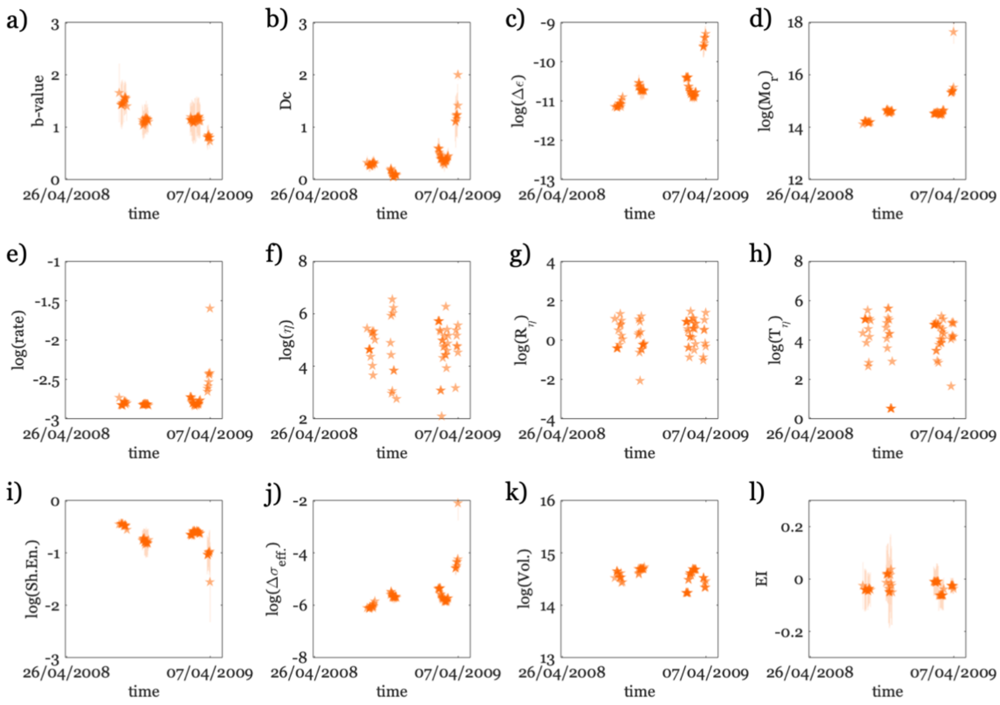

Figure S8. The same as Figure S6, but considering a cutoff magnitude equal to Mw 1.9

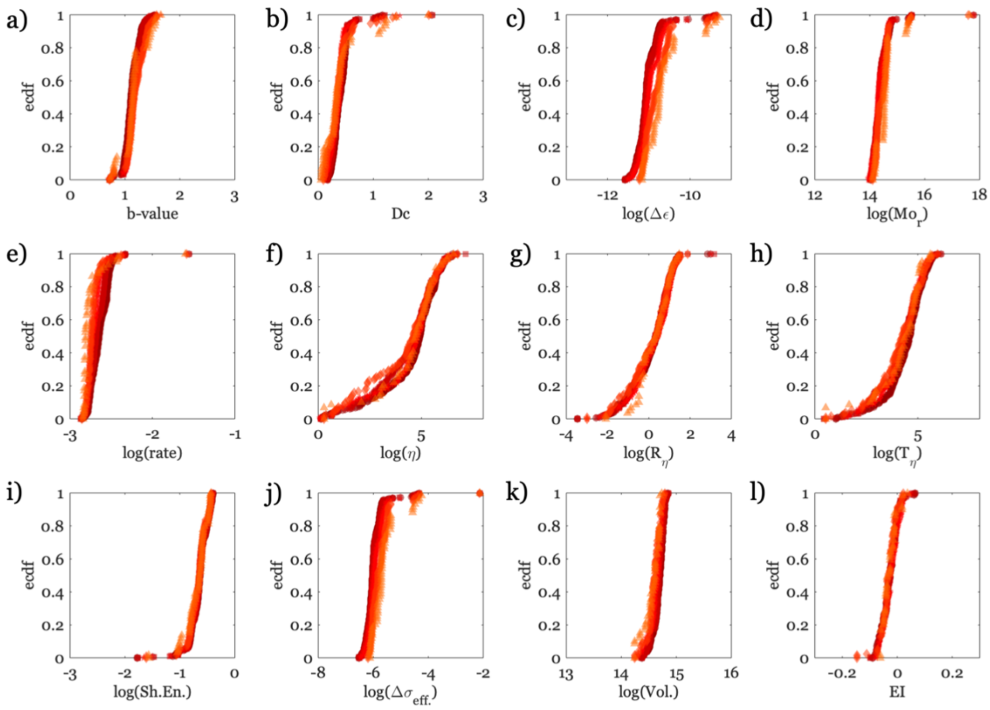

Figure S9. ECDF obtained for the 12 features considering different cutoff magnitudes: Mw 1.5 (dark red), Mw 1.6 (red), Mw 1.7 (light red), Mw 1.8 (orange), Mw 1.9 (yellow).

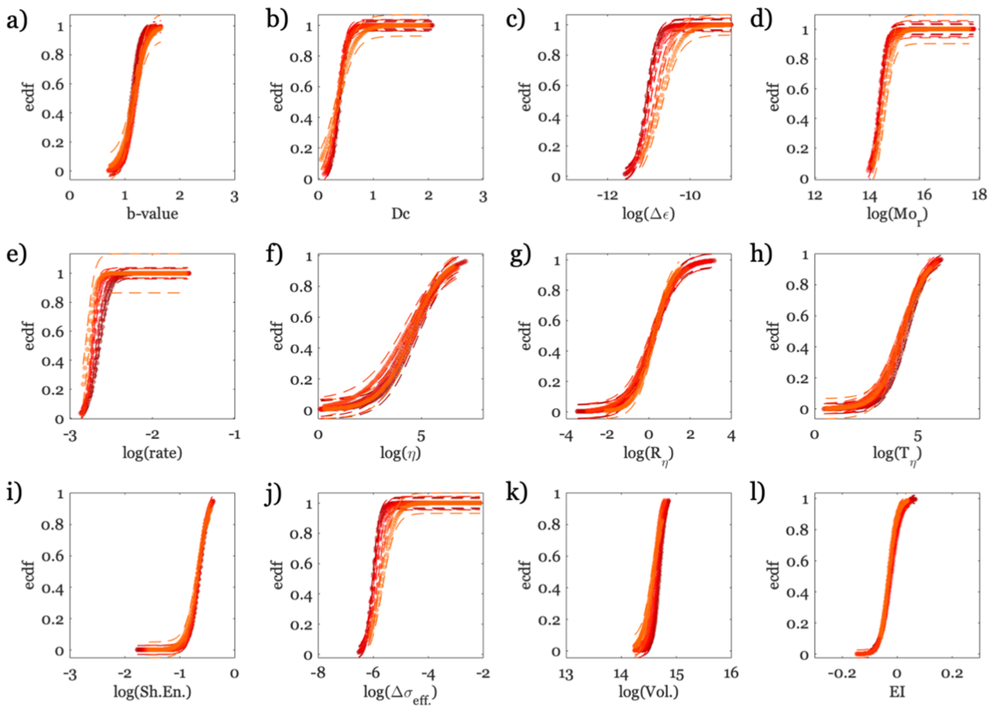

Figure S10. The same as Figure S11, but for the CDF obtained fitting the ECDF. For each features and cutoff magnitude we show the best model (colored line) +/- 1 standard deviation (dashed lines).

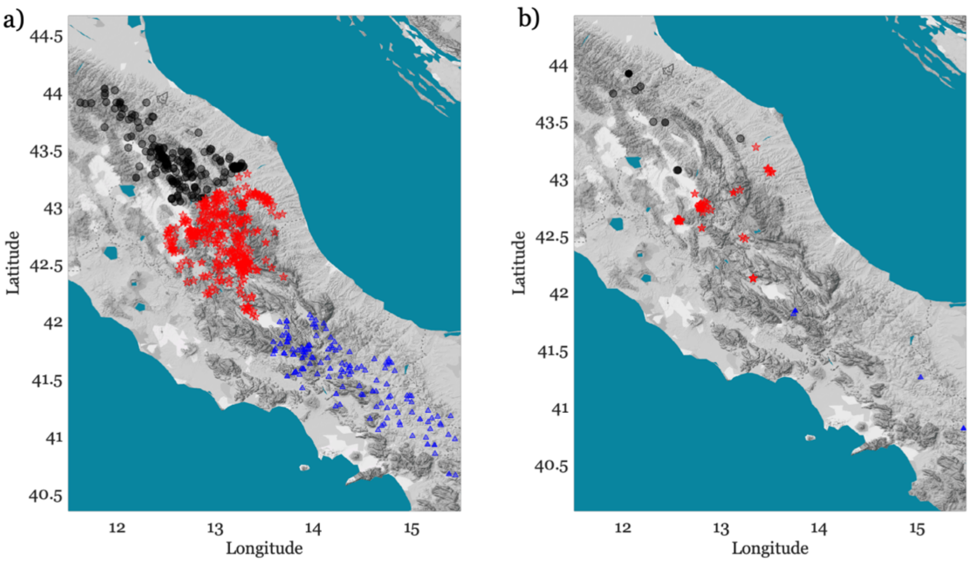

Figure S11. a) Distribution of earthquakes belonging to the background family and cutoff magnitude Mw 1.5, associated to the northern (black), central (red) and southern (blue) clusters. b) The same as a), but for the clustered seismicity.

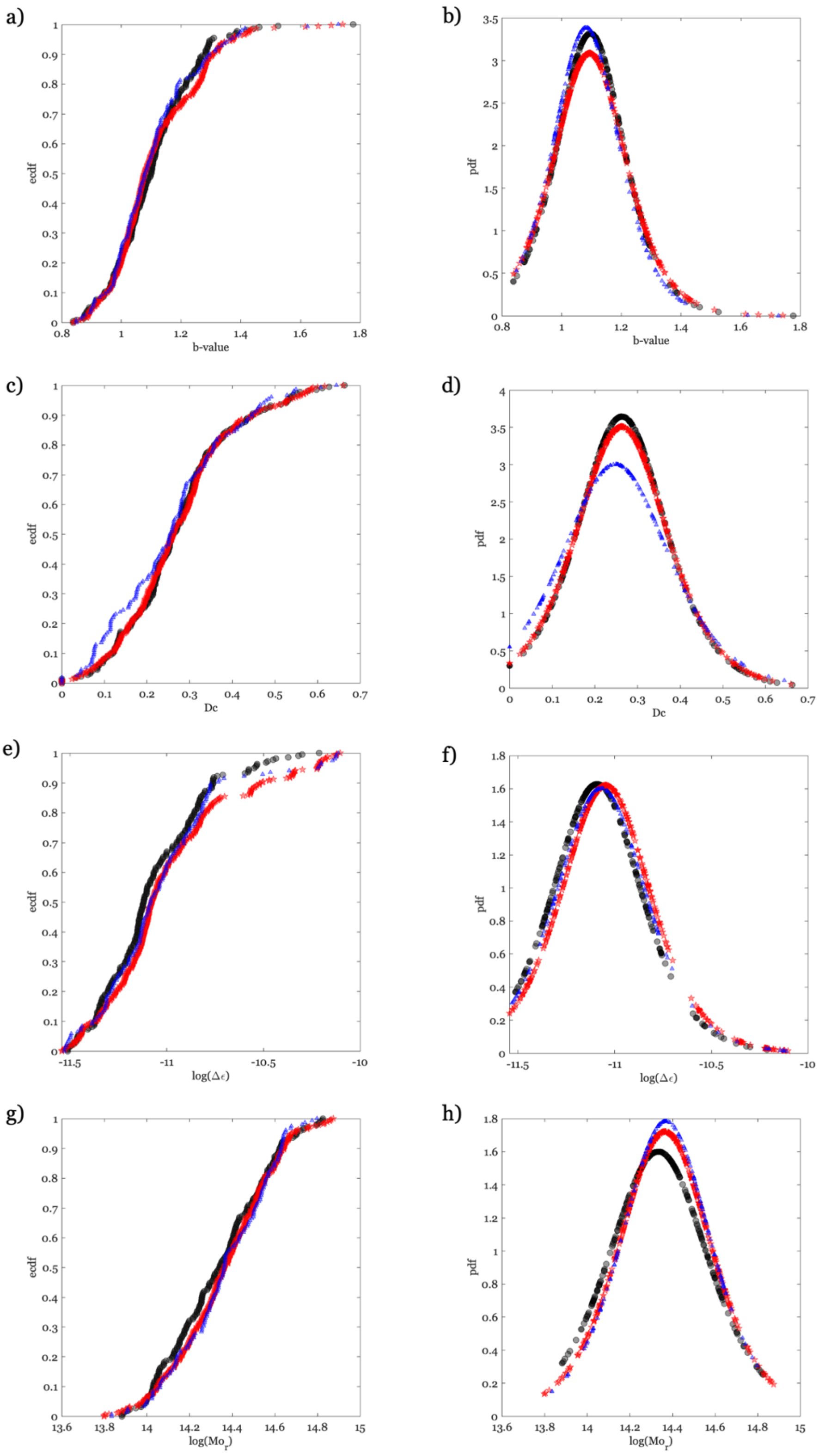

Figure S12. Left column subplots) ECDF for *b, Dc,* $\Delta\varepsilon$, $\dot{M}_0$ and associated to the northern (black), central (red) and southern (blue) clusters (see Figure S11). Right column subplots) The same as the Left column subplots, but for the PDF.

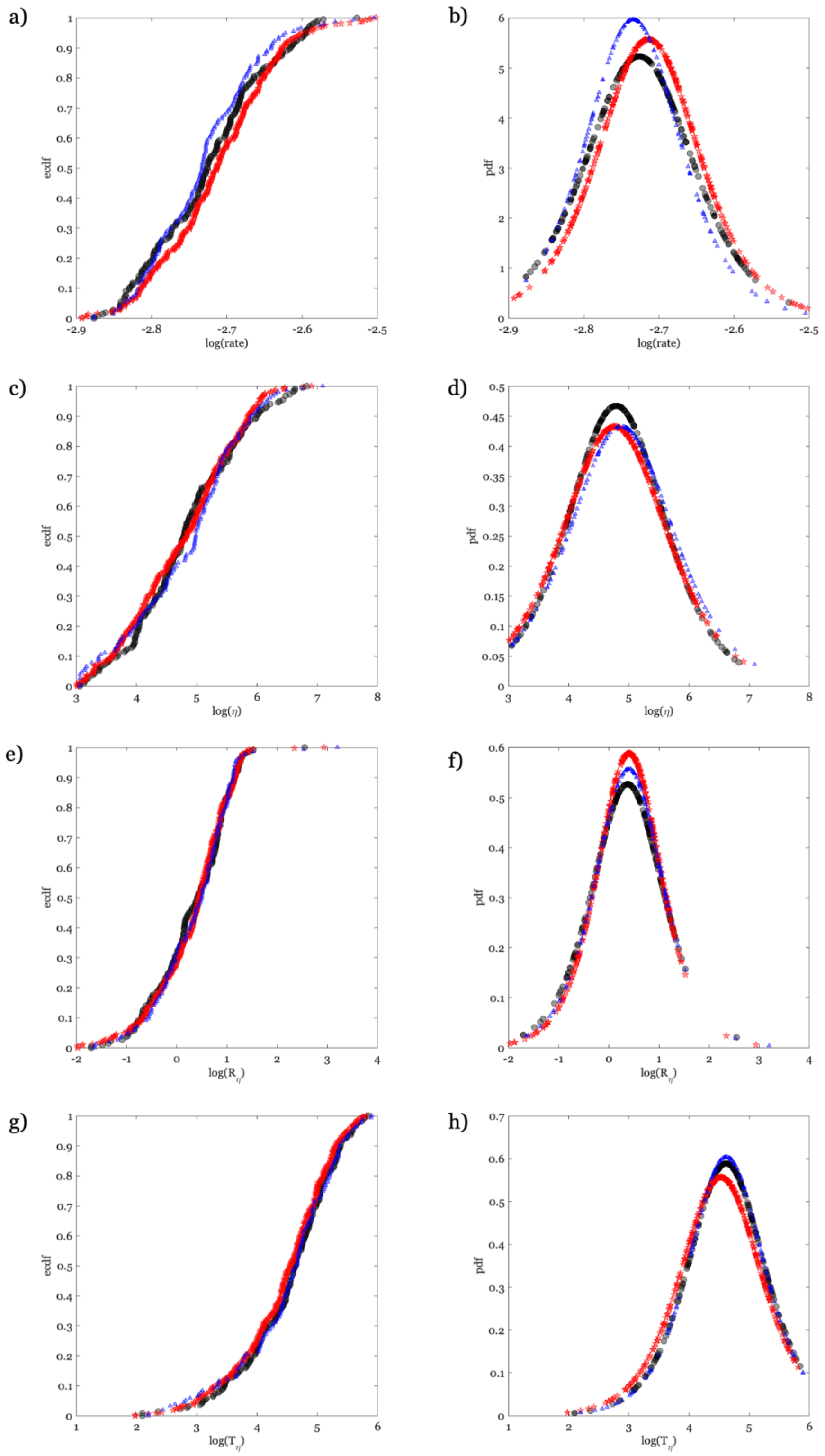

Figure S13. The same as Figure S12, but for ρ, η, $R\eta$, $T\eta$.

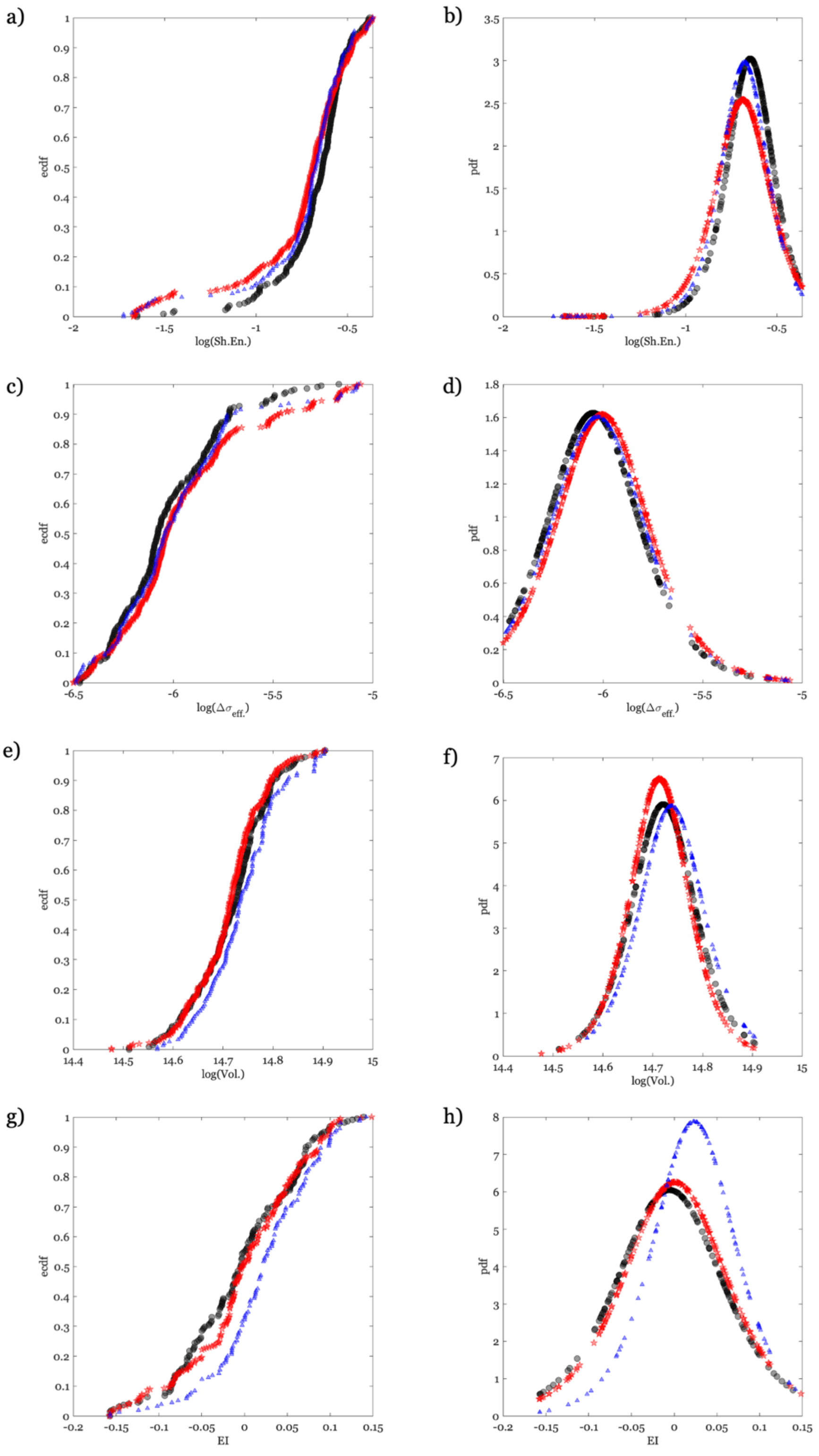

Figure S14. The same as Figure S12, but for H, $\Delta\sigma_e$, V, EI.

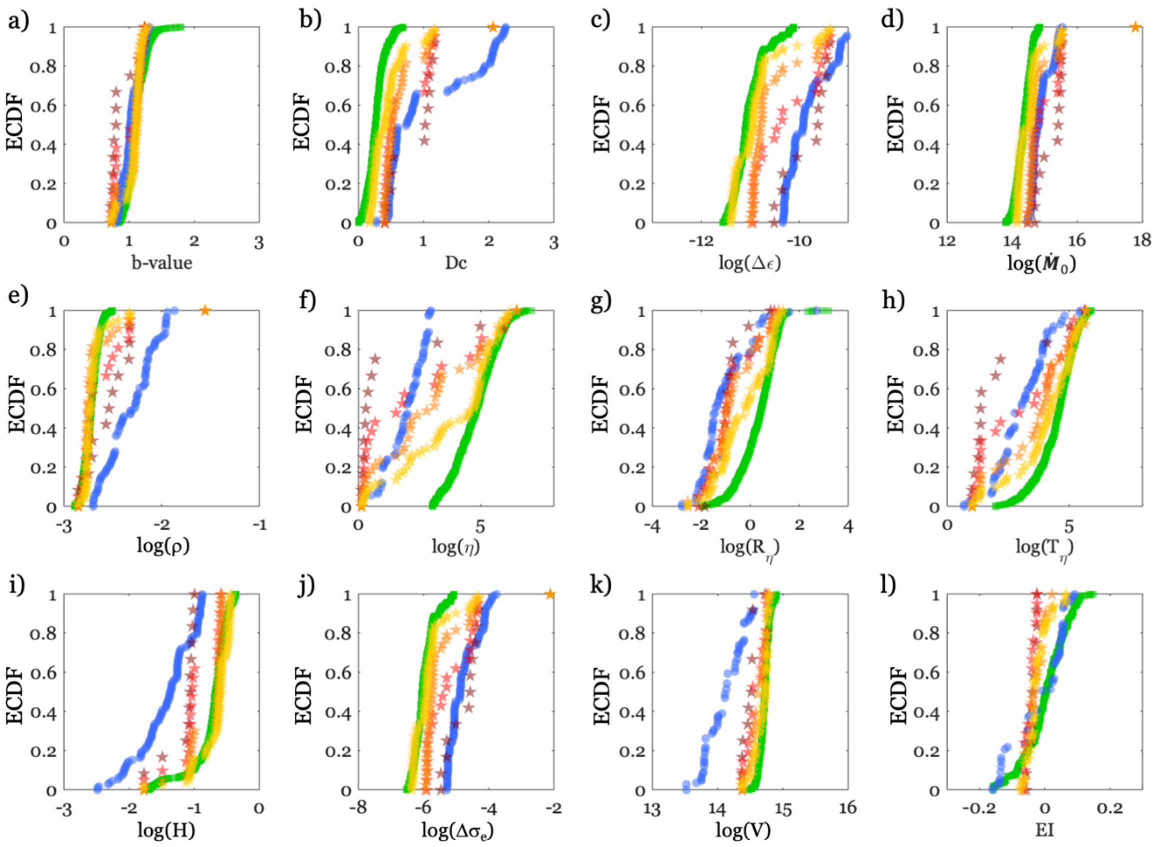

Figure S15. Empirical Cumulative Density Function (ECDF) computed for the different features and seismicity belonging to different periods. From (a) to (l) we show for each feature the ECDF for the background seismicity in green, the clustered one in blue, and those of different time periods before the Mw 6.3 L'Aquila earthquake (120 days yellow, 60 days orange, 30 days light red, 15 days dark red).

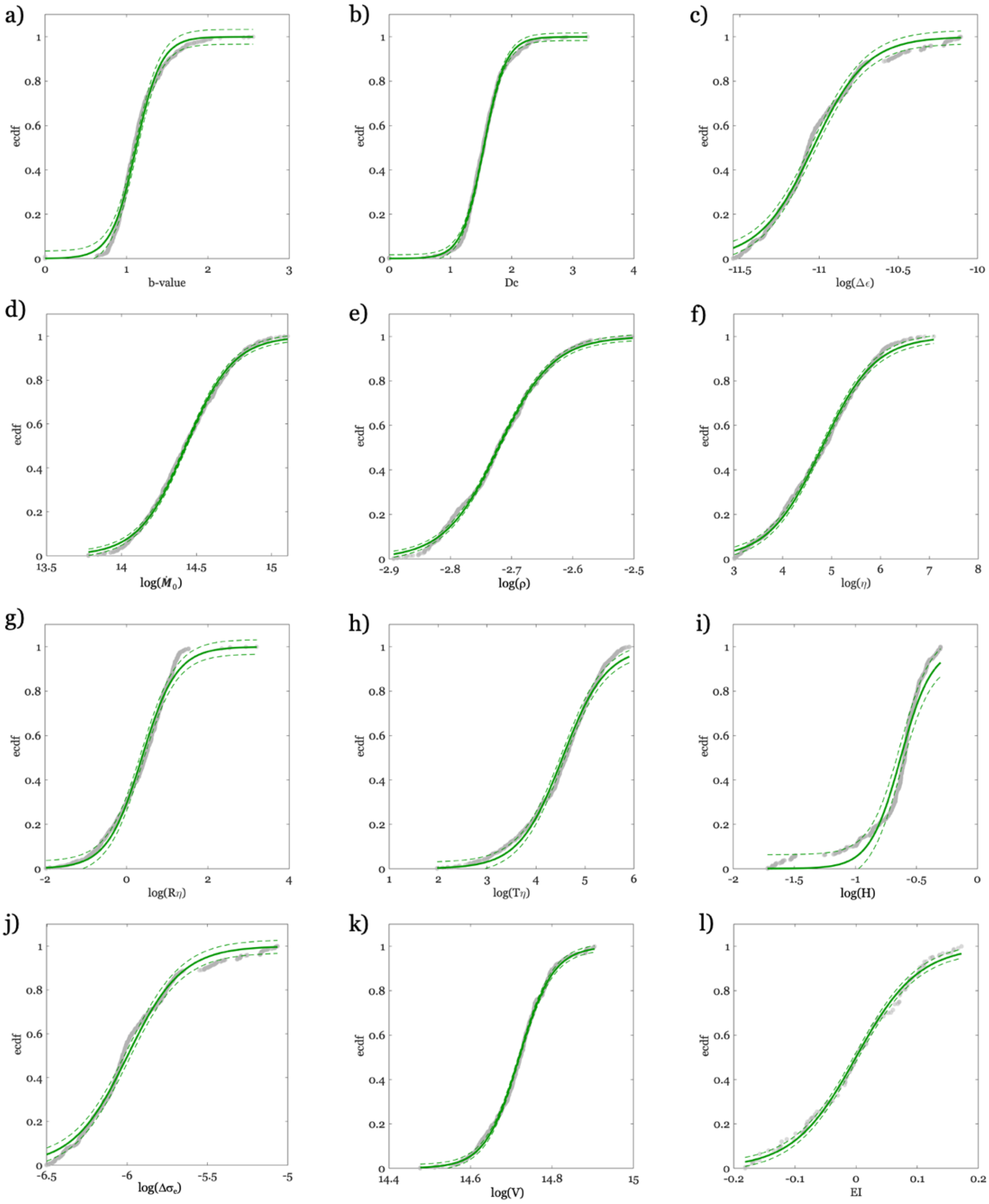

Figure S16. Fit of the ECDF for background seismicity with logistic functions. From (a) to (l), ECDF for the different features (gray dots), best-fit logistic function (green line) +/- 1 st.dev. (green dashed line).

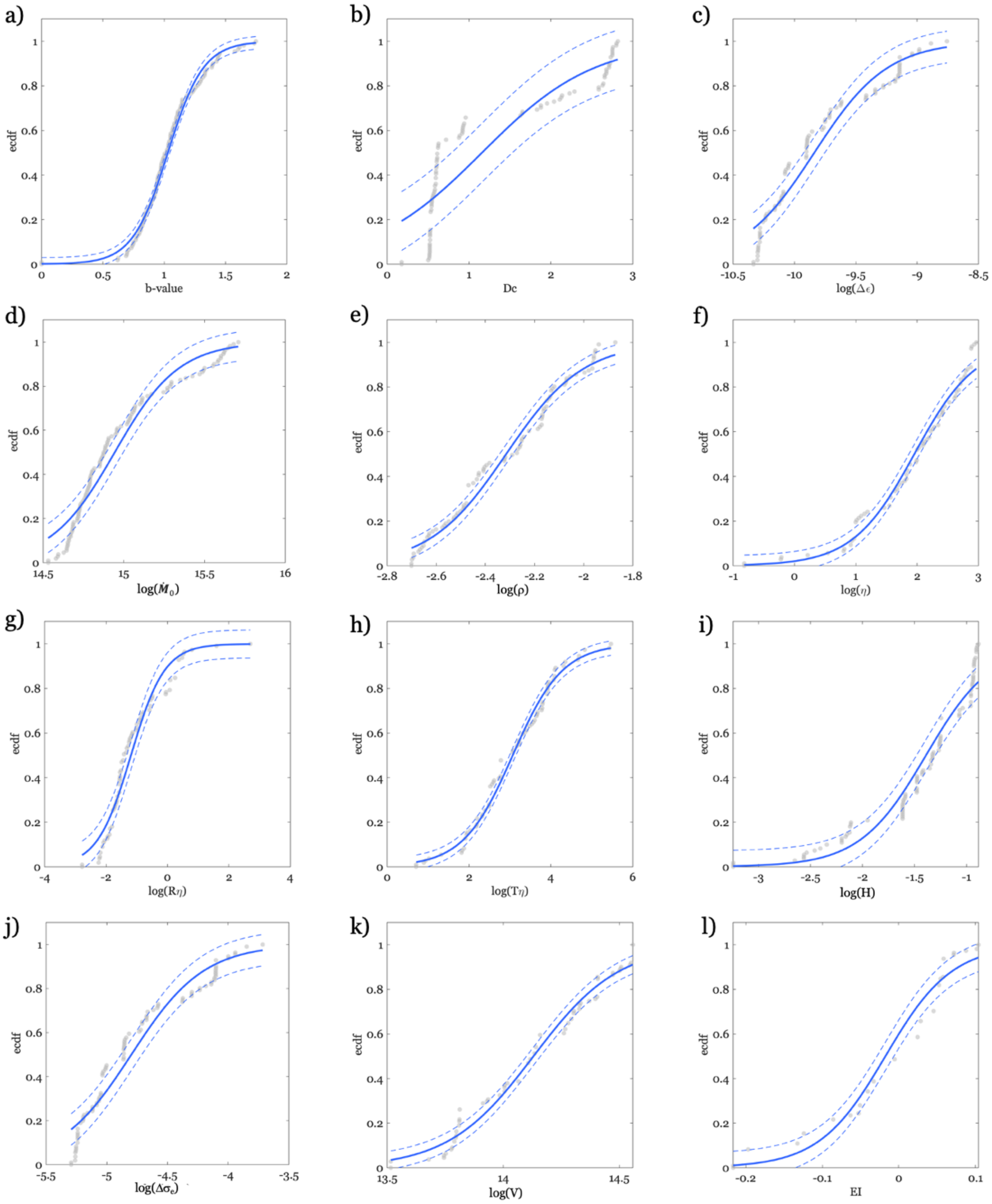

Figure S17. Fit of the ECDF for clustered seismicity with logistic functions. From (a) to (l), ECDF for the different features (gray dots), best-fit logistic function (blue line) +/- 1 st.dev. (blue dashed line).

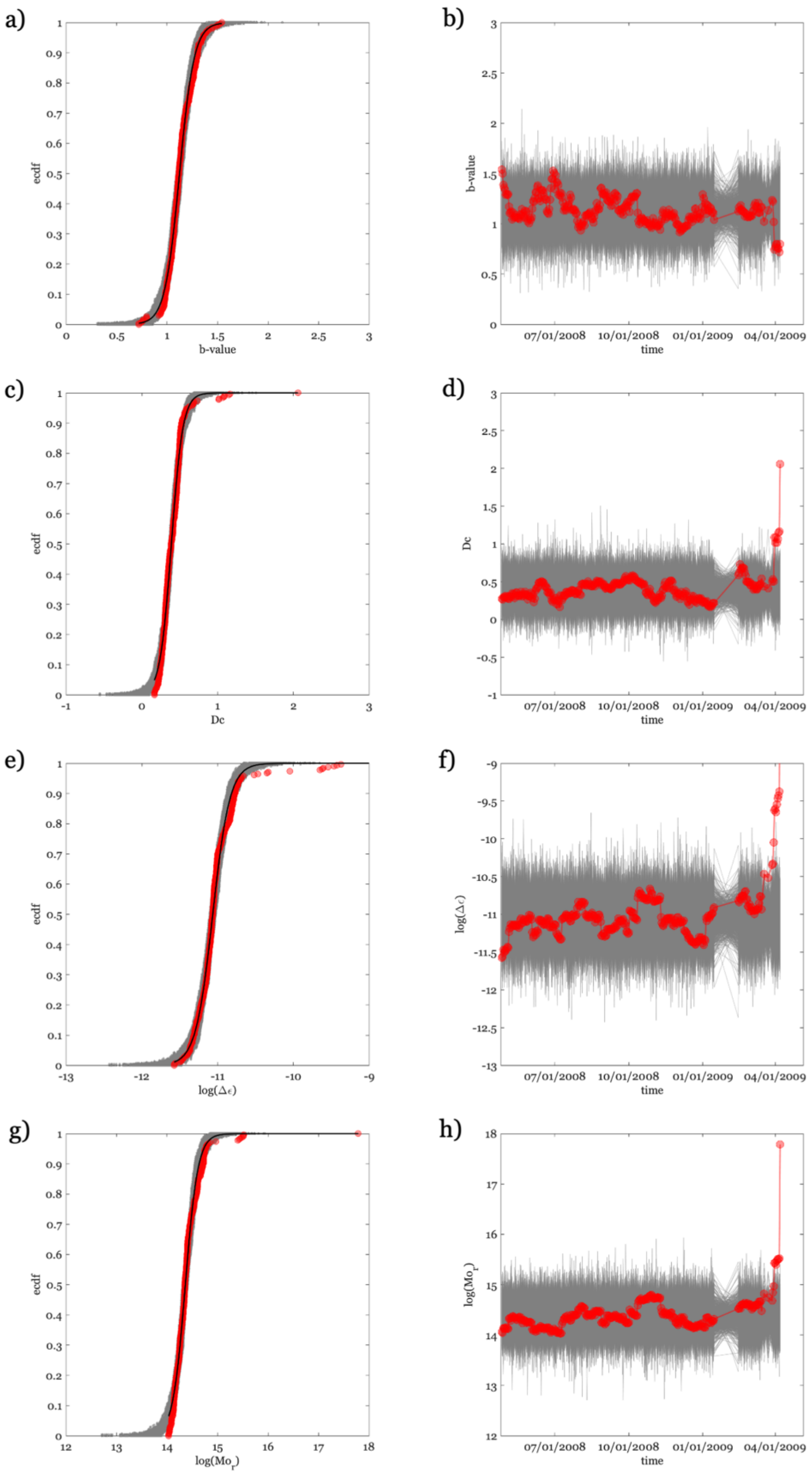

Figure S18. Monte Carlo analysis result for *b, Dc,* $\Delta\varepsilon$, $\dot{M}_0$. Left column subplot) ECDF for AQU09 (red), best fit model (black), and random sets of value (gray). Right column subplots) Time series for the random values (gray) and AQU09 (red).

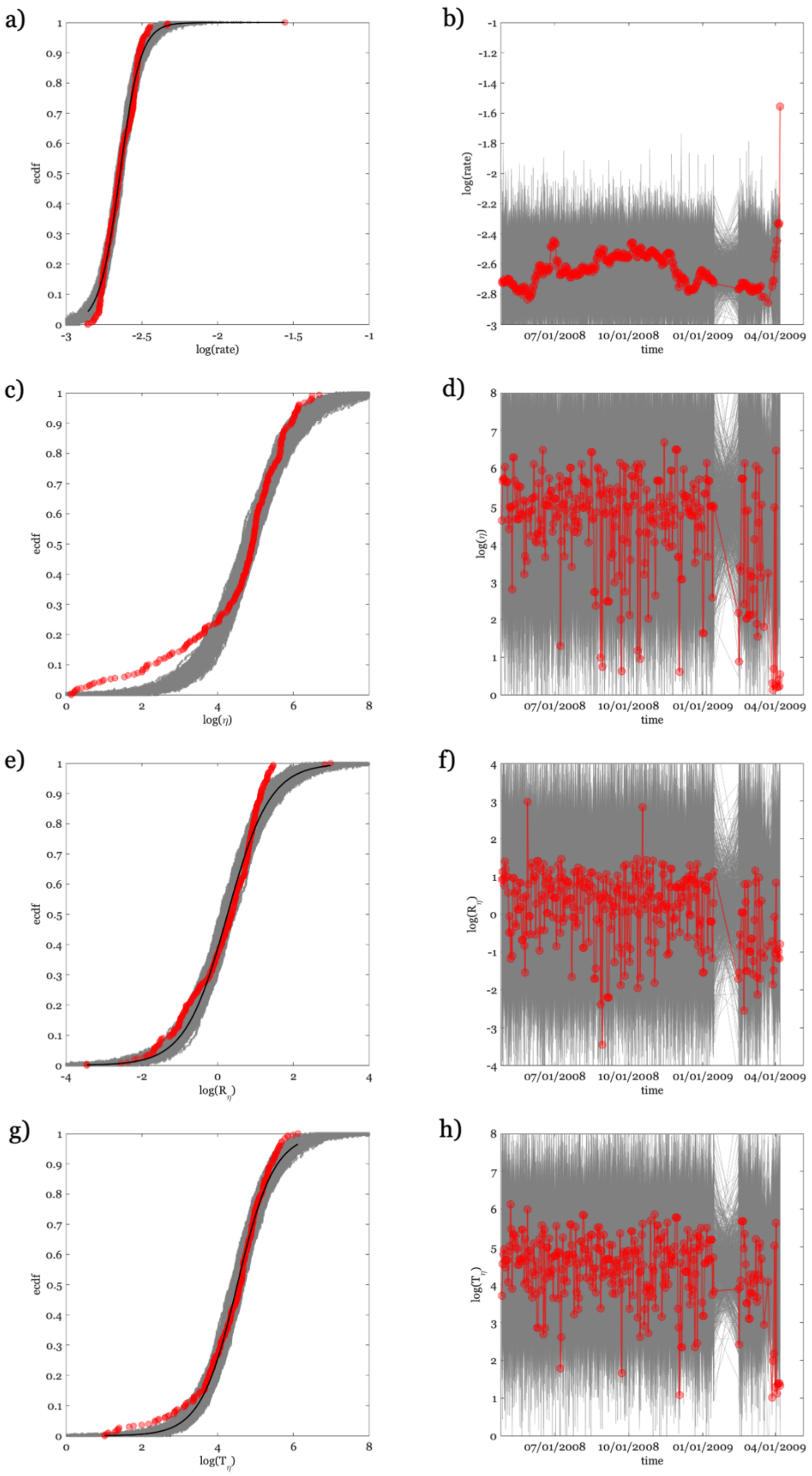

Figure S19. The same as Figure S18, but for ρ, η, $R\eta$, $T\eta$.

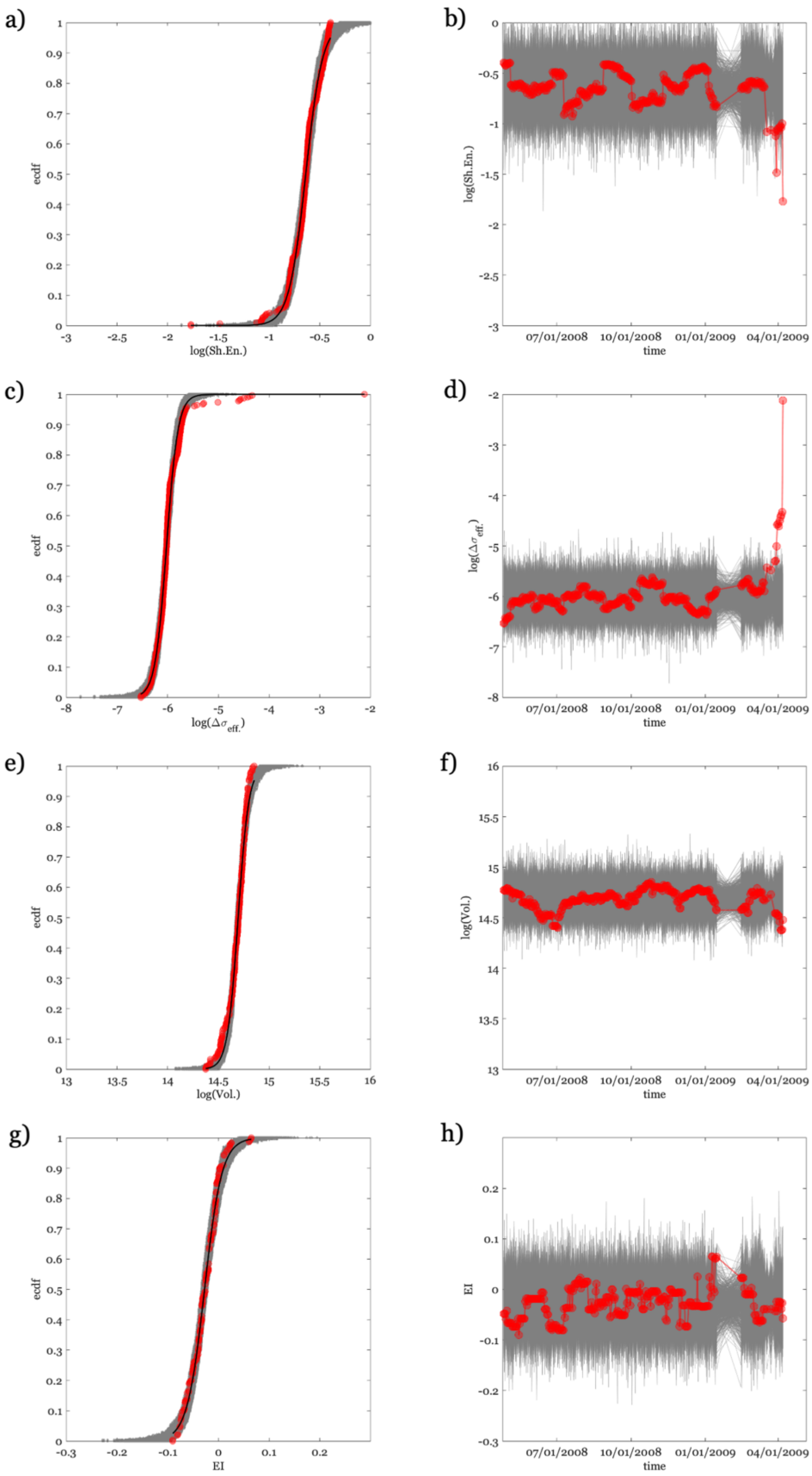

Figure S20. The same as Figure S18, but for H, $\Delta\sigma_e$, V, EI.

Table S1: SNR values estimated for the single features by a Monte Carlo approach and their mean (see comment above).

	b	Dc	$\Delta\varepsilon$	$\dot{\boldsymbol{M}}_0$	ρ	η	$R\eta$	$T\eta$	H	$\Delta\sigma_e$	V	EI	Mean
SNR-last value	0.031	0	0	0	0	0	0.079	0.05	0	0	0.035	0.19	0.032
SNR-Slop	0.003	0	0	0.005	0.002	-	-	-	-	0	0.065	-	0.011
